# Effects of Polypropylene and Polyethylene Terephthalate Microplastics on Trypsin Structure and Function

**DOI:** 10.3390/ijms26135974

**Published:** 2025-06-21

**Authors:** Tamara Lujic, Nikola Gligorijevic, Dragana Stanic-Vucinic, Maja Krstic Ristivojevic, Tamara Mutic, Lukas Wimmer, Lea Ann Dailey, Tanja Cirkovic Velickovic

**Affiliations:** 1 University of Belgrade—Faculty of Chemistry, Studentski trg 12-16, 11000 Belgrade, Serbia; lujict@chem.bg.ac.rs (T.L.); dstanic@chem.bg.ac.rs (D.S.-V.); krstic_maja@chem.bg.ac.rs (M.K.R.); tmutic@chem.bg.ac.rs (T.M.); 2 University of Belgrade—Institute of Chemistry, Technology and Metallurgy, National Institute of the Republic of Serbia, Njegoseva 12, 11000 Belgrade, Serbia; nikola.gligorijevic@ihtm.bg.ac.rs; 3 University of Vienna, Department of Pharmaceutical Sciences, Josef-Holaubek-Platz 2, 1090 Vienna, Austria; lukas.wimmer@univie.ac.at (L.W.); leaann.dailey@univie.ac.at (L.A.D.); 4 University of Vienna, Doctoral School of Pharmaceutical, Nutritional and Sport Sciences, Josef-Holaubek-Platz 2, 1090 Vienna, Austria; 5 Serbian Academy of Sciences and Arts, Knez Mihailova 35, 11000 Belgrade, Serbia

**Keywords:** microplastics, trypsin, polypropylene, polyethylene terephthalate, adsorption, soft corona, hard corona, meat extract proteins, α-Gal carrying proteins

## Abstract

Ingestion is one of the main exposure routes of humans and animals to microplastics (MPs). During digestion, MPs can interact with both gastrointestinal enzymes and food proteins. This study investigated the adsorption of trypsin onto polypropylene (PP) and polyethylene terephthalate (PET) MPs, the influence of MPs on trypsin structure and activity, and the in vitro trypsin digestibility of bovine meat extract (BME) sarcoplasmic proteins and BME α-Gal-carrying allergens (α-GalA) in the presence of PP and PET MPs. Trypsin, BME and α-GalA proteins interact with MPs, resulting in the formation of a soft (SC) and hard (HC) corona. This interaction is dynamic, leading to the adsorption and desorption of protein through time. Trypsin adsorption onto MPs results in slight structural changes in the SC and bulk solution, while a trypsin fraction residing in the HC loses most of its specific activity. The presence of MPs slightly slows down the digestibility of proteins with a mass of 38 kDa, while it does not affect the digestion of α-GalA. According to our results, it is unlikely that realistic concentrations of MPs in the intestine would have significant effects on meat extract proteins’ and allergens’ digestibility by trypsin. We confirmed that during trypsin digestion, the corona on PP and PET MP is composed of BME sarcoplasmic proteins and allergenic α-Gal-carrying proteins.

## 1. Introduction

Microplastics (MPs) have become a well-known pollutant in the environment, particularly due to the increasing production of plastic waste. The most common definition of MPs is that they are plastic particles greater than 1 µm and less than 5 mm in size [1]. MPs have been found in a variety of foods, including seafood, such as fish, shellfish, seaweed, packed meat, chicken gizzard, crop, fruits and vegetables, seaweeds, salt, honey, sugar and beverages, such as beer, nonalcoholic beverages and milk, as well as in tap and bottled water [2,3,4,5,6]. While studies on the presence of MPs in livestock are still lacking, MPs have been found in chicken gizzards because of trophic transfer [7]. The main sources of MPs in food are food processing and packaging [8,9,10,11].

The possible exposure routes of MPs to humans are ingestion, inhalation, and dermal penetration, whereby ingestion is considered as the primary route. Exposure levels of MPs per capita per day are estimated to be 553 particles (184 ng/capita/day) and 883 particles (583 ng/capita/day) for children and adults, respectively [12,13]. Several studies have reported the presence of microplastics in almost all human stool samples [14,15,16] including in children [17] and infant feces [18]. Moreover, microplastics were also found in the human stomachs of 26 cadavers [19] as well as in human colons from surgery samples [20,21]. Although MPs have long been considered inert, there is evidence of the impact of MPs on health. In the digestive tract, MPs have been linked to gut dysbiosis, inflammation, intestinal obstruction and mechanical damage, oxidative stress, and gut barrier destruction [22].

Due to their large surface area, MPs can interact and bind with different molecules present in the environment, including biomolecules. The mechanism of binding of biopolymers is proposed to be multilayered, with a formation of soft and hard coronae [23]. However, there are only three studies investigating the digestion of biomolecules in the presence of MPs on a molecular level. Tan et al. [24] showed that lipid digestion is reduced in the presence of MNPs (size range from 50 nm to 50 µm) due to decreased lipid bioavailability, as well as the binding of lipase, resulting in its reduced activity through conformational changes. Similar findings were made by Zhu et al. [25] while exploring the effects of three sizes of polystyrene MPs (30, 50 and 100 µm) on soybean oil digestion. In another study, it was found that pepsin, the main protease in the stomach, can bind to polystyrene MPs (10 µm), leading to a decrease in its activity, and that the presence of MPs influences pepsin activity and milk protein digestion by pepsin [26].

Food allergens are antigens triggering an allergic response by IgE binding after initial sensitization, and one of the main characteristics of proteins triggering an allergic response via the gastrointestinal tract is resistance to gastrointestinal digestion [27]. Although the relationship between MPs and food allergies is not yet fully understood, MPs might contribute by binding to food allergens and inducing structural changes, modifying the digestibility of these allergens, increasing intestinal permeability, promoting an inflammatory gut environment, and causing intestinal dysbiosis [28]. Furthermore, a recent study has shown that the “protein coronae” formed by MPs and the *Platanus acerifolia* pollen allergen Pla a3 caused more damage to A549 cells than Pla a3 alone, due to an increase in oxidative stress and cytokine production [29]. However, publications demonstrating the effects of MPs on the digestion of food allergens are still scarce [26,30].

The α-Gal syndrome (AGS) represents a severe form of delayed allergy to mammalian meat, and is an increasingly recognized public health issue. AGS manifests as a delayed early-phase allergic response, typically occurring 2–6 h post-consumption, triggered by IgE antibodies targeting the oligosaccharide galactose-α-1,3-galactose present in glycoproteins and glycolipids of mammalian meat (such as pork, beef, or lamb) or dairy products [31].

Trypsin is the main protease present in the small intestine, with a well-understood mechanism of action. It is a serine protease that specifically cleaves peptide chains at the carbonyl end of positively charged amino acid groups, such as lysine and arginine. There are several studies investigating the effects of ingested polystyrene (PS) and polyvinyl chloride (PVC) MPs on trypsin activity in marine and freshwater organisms, whereby decreased [32,33,34] or increased trypsin activity [35,36] was observed. In addition to impacting trypsin activity, the same studies found that MPs can also affect the activity of other pancreatic enzymes, such as amylase, lipase [32,34,35], chymotrypsin [32,34,35,36] and carboxypeptidase A [35], but with mixed results. The stimulation of pancreatic enzymes’ activity by the presence of MPs in the gastrointestinal tract (GIT) was attributed to compensatory secretory responses to improve digestion and absorption [36]. However, there is only one study showing the direct effects of MPs on trypsin activity. Liu et al. [37] investigated the adsorption of trypsin on PVC NPs (0.8 µm in size), showing that this interaction affected trypsin structure with limited influence on its activity. Currently, there are no data from the literature on the direct effects of PET and PP MPs on the structure and function of trypsin, an important intestinal digestive enzyme.

This study aimed to investigate the effects of the presence of two types of MPs, polypropylene (PP, 117 and 326 μm) and polyethylene terephthalate (PET, 55–63 μm), at a concentration of 20 mg/mL, often found in food and water [4], on trypsin structure and activity, as well as on the in vitro digestibility of proteins and allergens from beef meat extract by trypsin. The adsorption of trypsin onto the surface of MPs was examined by the analysis of adsorption isotherms and the formation of trypsin hard and soft coronae. The influence of trypsin adsorption on its secondary and tertiary structure was revealed by CD spectrometry, while the effects on activity were determined using a small synthetic substrate, Nα-Benzoyl-DL-arginine 4-nitroanilide hydrochloride (BAPNA). The in vitro digestibility of bovine meat extract (BME) sarcoplasmic proteins, as well as their soft and hard corona formation on the surface of MPs during digestion, were analyzed by SDS PAGE. Additionally, the digestibility and soft and hard corona formation of α-Gal-carrying meat allergens were analyzed by the immunoblot detection of the α-Gal moiety. This study provides further insight into MPs’ interactions with the GIT enzyme and the impact that this interplay can have on protein digestion, including for food allergens.

## 2. Results

### 2.1. Binding Analysis of Trypsin to MPs

The binding of trypsin to MPs was analyzed by adsorption isotherms, and it was demonstrated that the equilibration time of trypsin and PET is 1 h (Figure S1); hence, this time point was further used for the determination of adsorption parameters (Table 1) by fitting the obtained data into four binding isotherms (Figure S2). Even during mixing, reliable equilibration could not be obtained due to the floating of PP MPs in aqueous media. Thus, only adsorption onto PET MPs was analyzed.

The non-linear fitting of binding isotherms gave the best results for PET MPs with the highest R^2^. The affinity of trypsin for PET was relatively high, with adsorption capacities of 4.16 mg/g and 7.15 mg/g according to Langmuir and GAB models, respectively. The values obtained after fitting data into the Freundlich isotherm imply that the trypsin–PET mixture is a heterogeneous system, most likely due to multilayer adsorptions, and that trypsin is favorably adsorbed onto PET (1 < n < 10). The R_L_ value in the Langmuir isotherm equilibrium parameter is between 0 and 1, also implying favorable adsorption, but its low value indicates that adsorption leans towards the irreversible. The relatively high K_hard G_ in the GAB isotherm, which is the adsorption constant for the hard corona, also indicates strong binding in the hard corona, and low desorption. These results demonstrate that trypsin binds to the surface of PET MPs, with particularly strong binding in the hard corona.

### 2.2. Soft and Hard Corona Formation by Trypsin onto the Surface of MPs

**Figure 1:**
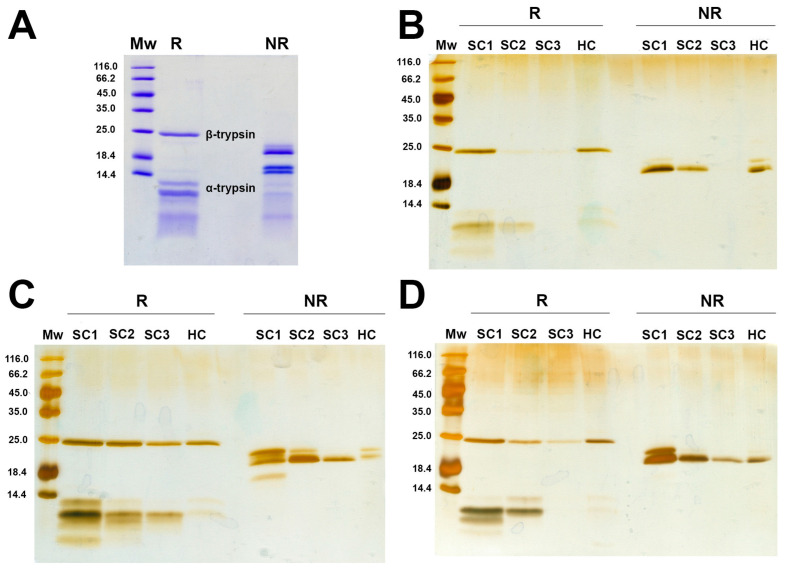
Reducing and non-reducing SDS-PAGE analysis of trypsin preparation on 16% PAA gel (**A**) and soft and hard corona obtained after 4 h incubation of trypsin with PET (**B**), sPP (**C**) and lPP MPs (**D**) on 14% PAA gels. Mw—molecular weight markers, R—reducing conditions, NR—non-reducing conditions, SC—soft corona, HC—hard corona.

To investigate the formation of trypsin hard (HC) and soft coronae (SC) on PET and PP MPs, the MPs were separated after 4 h of incubation with trypsin, followed by soft corona isolation. Soft corona washings and the hard corona components were analyzed by reducing and nonreducing SDS PAGE (Figure 1). The commercial trypsin preparation used in this study showed several bands under both conditions, originating from trypsin proteoforms (Figure 1A). Trypsin exists in several proteoforms, and the commercial preparation of bovine trypsin contains about 60–70% β-trypsin, 15–20% α-trypsin, 5–10% ψ- trypsin, and the remaining are minor isoforms, including γ-, δ-, and ξ-isoforms [38]. β-trypsin is a single intact polypeptide chain, and its autolysis generates two polypeptide α-trypsin chains. Further, intra-chain autolysis results in pseudotrypsin (ψ-trypsin) with three chains interconnected by disulfide bonds [39].

Trypsin forms both SC and HC on the surfaces of both PET and PP MPs (Figure 1B–D). The profiles of trypsin proteoforms from the SC were similar to that of the native trypsin preparation, with α- and β-trypsin dominating, suggesting that almost all proteoforms are bound in the SC.

The specific surface area of the smaller PET MPs (55 μm) per MPs mass was more than one order of magnitude higher than those of PP MPs (117 and 326 μm), hence the PET MP samples exhibited a higher surface area available for binding compared to PP MPs. Despite this, trypsin adsorbed in higher quantities onto the more hydrophobic PP surface, which accord with the general experimental finding that in most cases the affinity of proteins to surfaces increases with increased surface hydrophobicity [40]. Also, adsorption was slightly higher for sPP with a higher surface per mass ratio than for lPP. Moreover, in the SC of sPP, there was a higher ratio of autolyzed proteoforms (α-, γ-, δ-, ξ-trypsin)/β-trypsin than in the SC of PET MPs, suggesting the higher affinity of autolyzed proteoforms for more hydrophobic MPs.

### 2.3. Structural Analysis of Trypsin After Incubation with MPs

**Figure 2:**
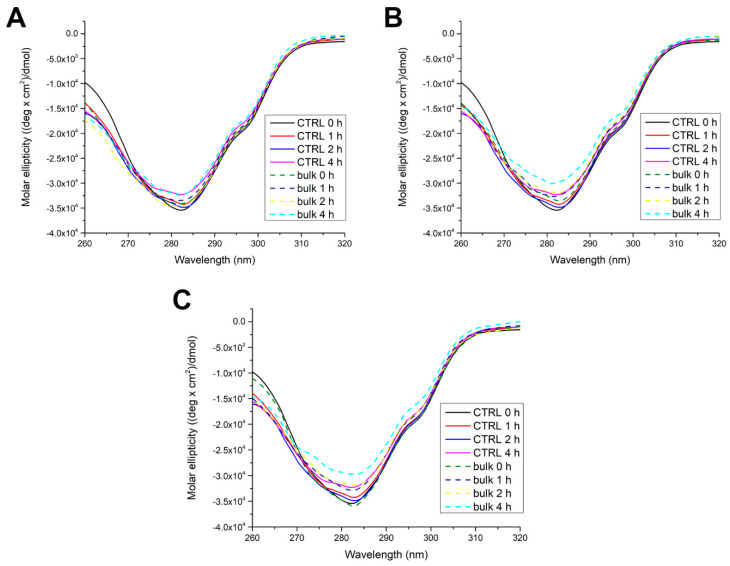
Near CD spectra of bulk trypsin after incubation without (CTRL—control) or with PET (**A**), sPP (**B**), and lPP MPs (**C**).

**Figure 3:**
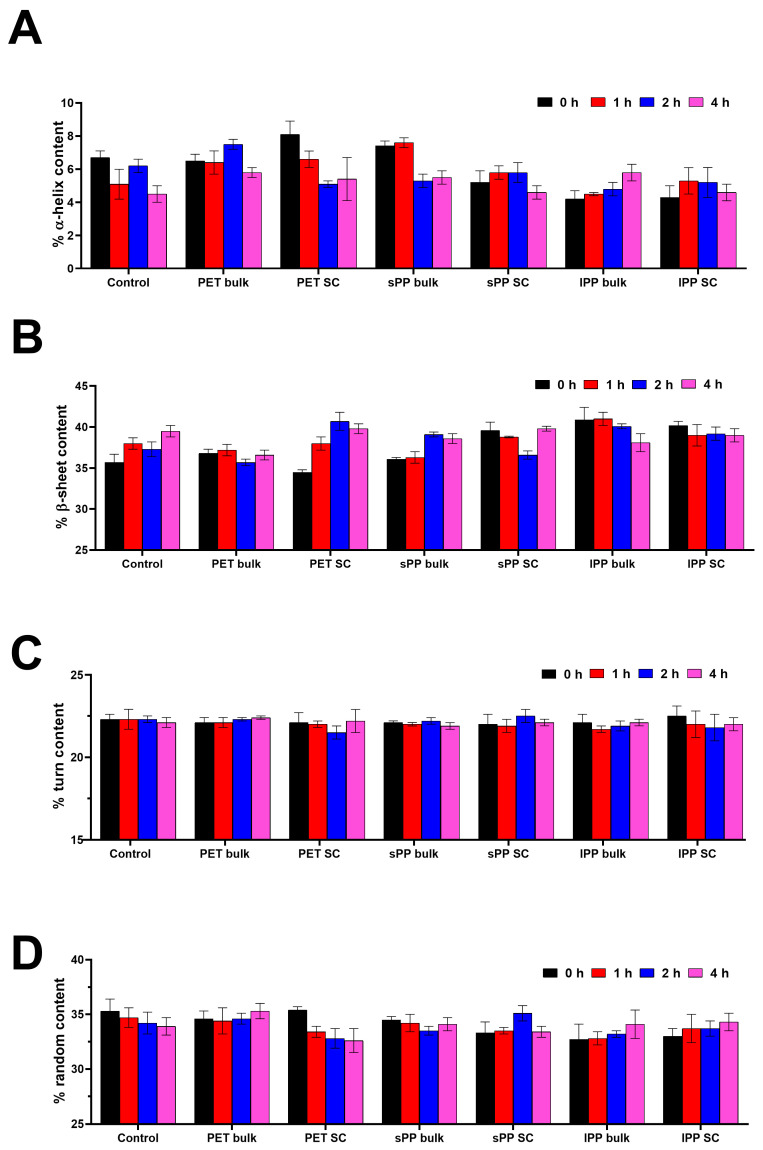
Content of secondary structures (%) in trypsin fractions (bulk and soft corona) after incubation without (control) or with PET and PP MPs—α-helix (**A**), β-sheet (**B**), turn (**C**) and random (**D**) (*n* = 4).

Further, we investigated the effects of protein adsorption onto the MP surface on the secondary and tertiary structures of trypsin. After incubating trypsin for up to 4 h with MPs, near (Figure 2) and far (Figure 3) UV CD spectra of separated bulk and soft corona trypsin solutions were recorded.

In the near CD spectra of native trypsin (control 0 h), a minimum at around 282 nm could be observed, originating from 8 Tyr and 4 Trp. Over time, the signal gradually decreased and was lowest after 4 h. This suggests that trypsin’s tertiary structure becomes slightly looser. In the presence of PET, trypsin’s tertiary structure behaves similarly to the control, having the highest flexibility after 4 h at the level of the control. The presence of sPP and lPP induced a gradual decrease in signal over time, but the CD signal after 2 h was at the level of the control after 4 h. These results imply that PP MPs of both sizes induced only a slight loosening of trypsin’s tertiary structure.

The far UV CD spectra of trypsin show one larger minimum at 208 nm and a smaller minimum at 222 nm, which is typical for porcine trypsin [41], indicating dominant β-sheet secondary structures (Section S2, Figure S3). The far UV CD signal of control trypsin decreased over time.

The calculation of secondary structure content provides deeper insights into secondary structure changes (Figure 3, Table S1 in Section S2). In comparison to the control, in the sPP SC, there is a slight decrease in α-helix content, and it is more pronounced for the lPP SC, while in the PET SC it was slightly increased. In bulk solutions, however, the α-helix contents in PET and sPP samples were similar to or higher than in the control. In the lPP sample, it was already lower after 5 min of incubation. In all SCs, the β-sheet content was slightly increased and became especially pronounced for lPP SC. In bulk solutions, the β-sheet content in PET and sPP samples was similar to in the control, and in the lPP sample it was higher. These results show that lPP has mostly an opposite effect on both α-helix and β-sheet contents in comparison to PET and sPP. These results suggest that lPP induced a slight α to β transition in both bulk and SC, whereas sPP induced it only in the SC. There were no differences in turn content. The random content in sPP bulk and PET bulk was similar to in the control, while it was slightly lower in all SC and lPP bulk samples, suggesting that in all SCs there was a slight increase in ordered structures.

### 2.4. Influence of MPs on the Activity of Trypsin

As adsorption-induced changes of enzyme structure are likely to affect enzymatic activity, we explored the extent to which trypsin adsorption onto MPs influenced activity in the bulk solution and in the adsorbed fractions (SC and HC). Trypsin was incubated with MPs for up to 4 h. After the separation of bulk solution and the isolation of SC, trypsin activity was determined in bulk solution, the SC and the HC corona.

#### 2.4.1. Influence of MPs on the Activity of Bulk and Soft Corona Trypsin

**Figure 4:**
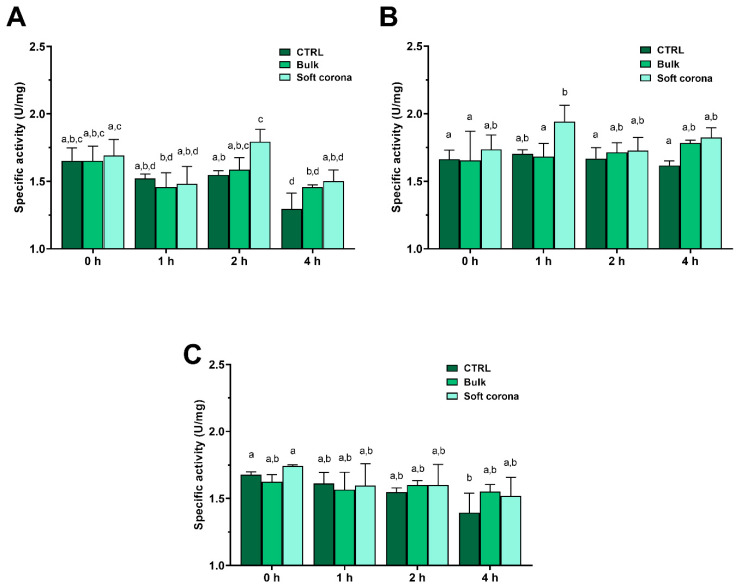
Specific activity of trypsin incubated without MPs (CTRL—control), and in bulk and the SC after incubation with PET (**A**), sPP (**B**), and lPP MPs (**C**). Different small letters denote statistical significance, i.e., if sample annotation does not contain the same letter, there is a statistically significant difference (*p* < 0.05) (*n* = 4).

In two out of three repeated positive controls (incubation of trypsin without the addition of MPs), the trypsin-specific activity decreased over time (Figure 4). This can be attributed to autolysis at low Ca^2+^ concentrations (0.6 mM), which is insufficient to stabilize the structure and prevent autolysis [42]. Following incubation with PET, there was a statistically significant increase in activity (*p* < 0.05) in the SC relative to the positive control after 2 h of incubation. Similarly, trypsin incubation with sPP demonstrated a statistically significant (*p* < 0.05) increase in activity in the SC after 1 h relative to the positive control after 0, 2 and 4 h, and bulk trypsin after 0 and 1 h. Following incubation with lPP, however, this difference was only statistically significant (*p* < 0.05) between the positive control after 4 h of incubation and at the 0 h time point. Compared to the control, the presence of MPs tended to preserve trypsin activity in the bulk solution and the SC after 4 h of incubation.

#### 2.4.2. Activity of Trypsin in the Hard Corona

After trypsin incubation with MPs for 1 h and the subsequent separation of bulk solution and soft corona, the trypsin-specific activity in the HC was determined. Moreover, the protein content in the HC after trypsin activity determination was semi-quantified by reducing SDS PAGE analysis, followed by densitometry.

**Figure 5:**
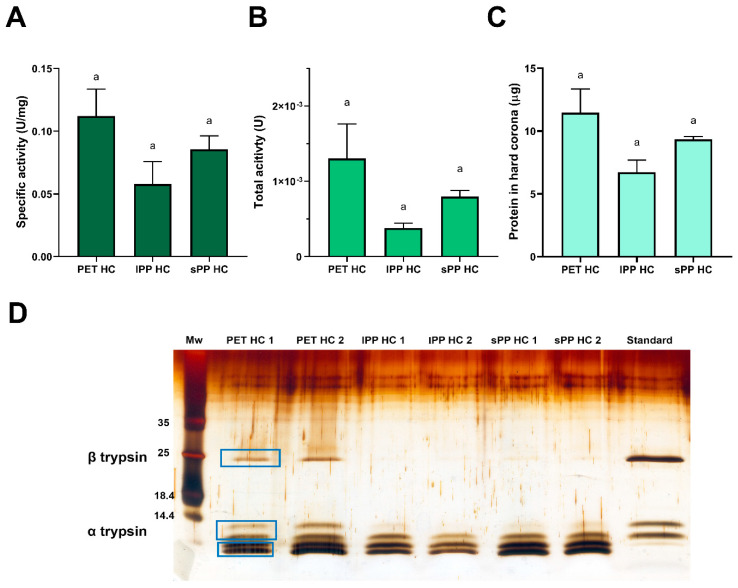
Trypsin-specific activity (**A**), total activity (**B**), estimated amount of protein (**C**) and protein profile of trypsin (**D**) bound in hard corona after 1 h of trypsin incubation with MPs (*n* = 2). The protein profile of trypsin was analyzed using SDS PAGE under reducing conditions after silver staining. The amount of protein in hard corona was estimated by densitometry via reducing SDS PAGE analysis. For the trypsin standard a preparation of 0.66 μg was applied.

The specific activity of trypsin in the HC after 1 h of incubation with MPs was an order of magnitude lower than the activity in the bulk or soft corona (Figure 5A). Similar results have been obtained before for trypsin that was irreversibly bound to polystyrene nanoparticles as a result of changes in both secondary and tertiary structures [43]. There is a trend towards a higher total trypsin activity in the PET HC compared to the lPP HC and sPP HC, although this is not statistically significant (Figure 5B). This is due to the tendency for binding of a higher amount of protein in the HC of PET than in sPP and lPP HC (Figure 5C), as well as due to a higher amount of more active α- and β-trypsin isoforms in PET HC, in comparison to both PP HCs (Figure 5D). After 1 h of MP incubation with trypsin, the amount of protein bound in the HC could be ranked as PET > sPP > lPP, in accordance with the surface area of MP available for trypsin binding (Figure 5C,D). As stated previously, a 1 h incubation of trypsin with PET MPs was enough to reach the adsorption equilibrium (Figure S1). However, this time seems to be longer for PP MPs, according to the hard corona profiles obtained after a 4 h incubation (Figure 1). After 1 h of incubation, there is a tendency toward a twofold increase in the ratio of specific activity in the SC and HC for sPP and lPP MPs compared to PET MPs (24 and 26 vs. 12.5, respectively). This demonstrates that the equilibrium between SC and HC was not reached after 1 h for PP MPs, resulting in lower amounts of trypsin in the PP hard corona, particularly of its active β and α proteoforms. Compared to the starting trypsin preparation, where the β and α-proteoforms are predominantly present, in the hard coronae there is a noticeable depletion of β- and α-proteoforms, as well as increases in peptides below the 14.4 kDa marker, most likely other less active autolytic proteoforms such as ψ-, γ- and δ-trypsin (Figure 5D). It is hypothesized that β-trypsin desorbs during incubation with BAPNA, while only traces of autolyzed proteoforms were desorbed due to their higher affinity to MPs. β-trypsin has a more compact hydrophobic core than α-trypsin [44], and it seems that the gradual loosening of this compact hydrophobic core with autolysis extent enables a higher affinity of autolyzed proteoforms towards the hydrophobic surfaces of MPs in comparison to β-trypsin (Section S1) (Figure 5D).

The content of trypsin in the HC represents only about 0.5% of total trypsin (0.57% for PET, 0.34% for lPP and 0.47% for sPP). This suggests that, taking into account total trypsin, the presence of MPs, even at the relatively high concentration of 20 mg/mL, exerted only a negligible effect on trypsin activity overall.

### 2.5. Influence of MPs on In Vitro Digestion of Meat Extract Proteins by Trypsin

Beef meat extract (BME), containing mostly sarcoplasmic proteins, was subjected to in vitro trypsin digestion in SIF with and without the presence of lPP and PET MPs. After digestion, the bulk solution was separated for all time points, and the SC was isolated. Bulk solutions, SC, and HC were analyzed by reducing SDS PAGE.

**Figure 6:**
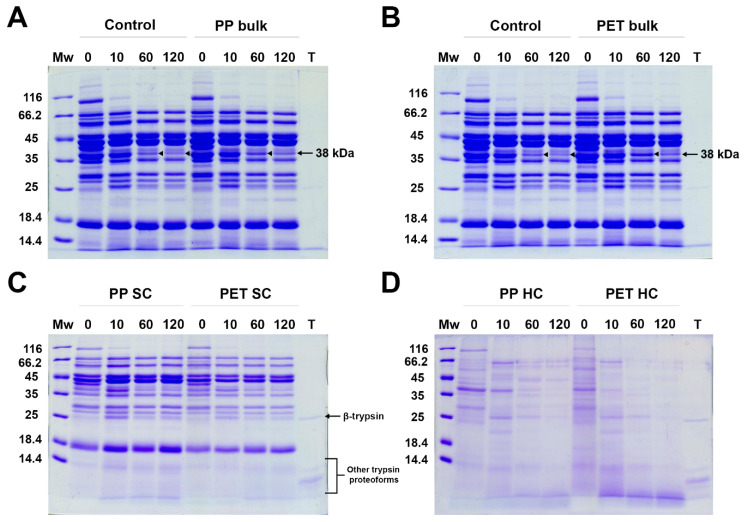
SDS PAGE analysis of BME proteins’ digestion by trypsin in SIF in the presence of MPs and without MPs (control). (**A**) Digestion of control and bulk solution in the presence of lPP MPs (lPP bulk). (**B**) Digestion of control and bulk solution in the presence of PET MPs. (**C**) Soft coronae (SC) of lPP and PET MPs during BME digestion. (**D**) Hard coronae (HC) of lPP and PET MPs during BME digestion; Mw—molecular weight markers, 0, 10, 60 and 120—digestion time in min, T—trypsin alone.

In general, incubation with PET and lPP MPs did not seem to have a substantial effect on the digestion rate of BME proteins with trypsin (Figure 6A,B). In all digestion mixtures, proteins around 150, 100, 40, 33 and 30 kDa were rapidly digested after 10 min, generating fragments of about 22 and 25 kDa. After 60 min, several more proteins were digested, including bands at 100, 60 and 38 kD, as well as generating a fragment at 25 kDa. After 120 min, only the band at 38 kDa was further digested in all samples. The intensity of the band at 38 kDa, however, was more intense in the presence of MPs than in the positive control, suggesting that the trypsin digestion of proteins within this band was delayed by the presence of both MPs.

At time point zero (0 min), BME proteins were actually in contact with MPs during the 5 min of centrifugation, but even this short time was enough for the meat proteins to form both an SC and an HC (Figure 6C). At this time point, the SC of both MPs was mostly a reflection of the most abundant protein present in the BME. However, after 10 min in the presence of PP, the SC protein bands were more intense than at 0 min, demonstrating the further adsorption of the most abundant proteins in the SC, including a fragment at 25 kDa generated by digestion. Even though PET MPs have a much larger available surface area, there was no further protein adsorption in the PET SC after 10 min, indicating that BME proteins exhibited a much higher affinity for PP than for PET MPs. Similar to the bulk solution, the SC fraction investigated after 60 min showed that bands at 100, 60, 38 kD and 25 kDa were also digested, implying that proteins residing within SC are prone to digestion at similar level as proteins in solution. Although the binding of β-trypsin cannot be judged due to the presence of protein/proteolytical fragments at 25 kDa, other trypsin proteoforms are poorly adsorbed in the SC.

In contrast to the SC, the 0 min HC time point for both mixtures with PP and PET MPs was dominated by proteins at about 30, 38, 70, 60 and 100 kDa. After 10 min, there were dominant bands at 60 and 38 kDa, as well as a fragment generated at 25 kDa, and these bands were more intense in the PP HC. Additionally, after 60 min and 120 min, a higher desorption of proteins from the PET HC than from the PP HC was observed, indicating a higher affinity to the PP surface rather than the PET surface. There were abundant short fragments (<5 kDa) in the PET HC, which were faint in the PP HC due to a much larger available PET surface area. Despite the fact that the contents of all proteins found in the SC were almost proportional to their concentrations in the bulk solution, the HC composition of tightly bound proteins appeared to also be determined by the protein affinity for the MP surface and the available surface area. Similar to the SC, the trypsin proteoforms seem to be poorly adsorbed in the HC. These results suggest that when in competition with a content of BME proteins that is about 50 times higher, trypsin adsorbs onto MPs only in trace amounts, and thus trypsin’s structure and activity is almost completely preserved. The observed slight decrease in the digestibility of proteins in the band of about 38 kDa may be a consequence of MPs affecting the structure of some proteins and their susceptibility to trypsin attack.

### 2.6. Influence of MPs on In Vitro Digestion of α-Gal Bearing Meat Allergens from Meat Extract by Trypsin

**Figure 7:**
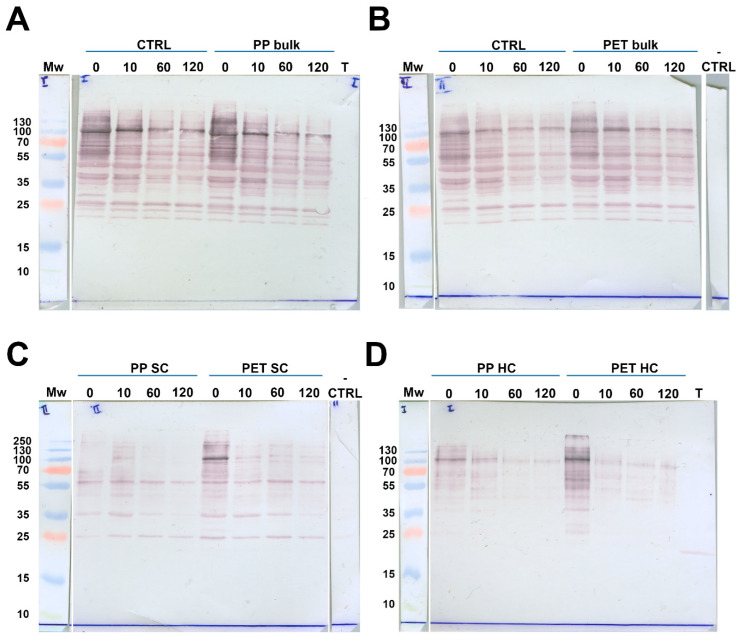
Immunoblot detection of α-Gal allergens from BME digested by trypsin in SIF in the presence and without MPs (control). (**A**) Digestion of control and bulk solution during digestion in the presence of lPP MPs. (**B**) Digestion of control and bulk solution in the presence of PET MPs. (**C**) Soft coronae (SC) of lPP and PET MPs during BME digestion. (**D**) Hard coronae (HC) of lPP and PET MPs during BME digestion. Mw—molecular weight markers;—CTRL—control for reactivity of secondary antibodies; 0, 10, 60 and 120—digestion time in min; T—trypsin.

Bulk solutions, SC, and HC were tested for the presence of proteins containing the α-Gal moiety by immunoblot. Even after 120 min of digestion without MPs (positive control), BME proteins were only partially digested, implying their considerable resistance to trypsin action. The α-Gal-carrying protein (α-GalP) profiles during digestion in the positive control sample were identical to profiles obtained during digestion with both PP and PET MPs, suggesting that the presence of MPs did not have a substantial effect on meat allergens (Figure 7A,B).

Similar profiles of α-GalP in bulk solutions and in the SC of both MPs during all digestion times demonstrated that proteins residing within the SC were prone to digestion at similar levels as proteins in solution (Figure 7). This was also observed for BME proteins. α-GalP also quickly formed an SC and HC after only 5 min of contact with the MPs (time point 0 min, Figure 7C,D). We also observed a higher abundance of α-GalP in the PET SC compared to the PP SC due to the larger available surface area. However, after 10 min, the PP SC protein bands were more intense than at 0 min, indicating the further adsorption of α-GalP. In the PET SC, there was pronounced protein desorption, showing higher α-GalP affinity to PP than to PET, as was also observed in the SC of total BME proteins.

In the PET HC at 0 min, α-Gal-carrying proteins were much more abundant than in the PP HC due to a much larger available surface area. After 10 min, the desorption of proteins from the HCs of both MPs was observed, but this was considerably more pronounced for those from the PET HC. This suggests a lower affinity of α-GalP for the PET than for the PP surface. These results demonstrate that α-Gal carrying proteins bind to both the SC and HC of both MP types, and that these proteins exhibited a higher affinity to PP than to PET, similar to total BME proteins.

## 3. Discussion

While several recent reviews cover the possible concerns related to and effects of micro- and nanoplastics on the GIT [45,46], studies on the direct interactions of food proteins and GIT enzymes with MPs, the effects of MPs on the structure and activity of GIT enzymes, as well as the influence of MPs on the digestibility of proteins within complex food matrices, particularly food allergens, are still scarce.

Trypsin’s adsorption to PET MPs was analyzed using four isotherm models, demonstrating that trypsin binds to the PET MPs surface, with particularly strong and favorable binding in the HC. In this study, the obtained Langmuir constant (10.15 mL mg^−1^) and Freundlich constant (4.08 (mg/g) × (mL/mg)^1/n^) were several times lower than those previously reported by Liu et al. [37]. For trypsin’s adsorption to PVC NPs with a size of 0.8 µm (Langmuir, 63.97 mL mg^−1^; Freundlich, 49.73 (mg/g) × (mL/mg)^1/n^), we can infer a lower affinity of trypsin to PET than to the PVC surface. In this study, a much lower adsorption capacity was obtained compared to that obtained by Liu et al. using the Langmuir model (4.16 vs. 51.16 mg/g). This is due to the substantially higher hydrophobicity of PVC over PET, with a two-fold higher LogP (SA)−1 value [47], as well as a much larger adsorption surface per MPs mass of 0.8 μm-PVC NP in comparison to the one for 55 μm-PET MPs used in this study.

Trypsin and its proteoforms were found in the soft and hard corona of MPs. Both intact β-trypsin and autolyzed forms were present at almost equal amounts in the SC. The quantity of bound protein seems to be higher for more hydrophobic surfaces (PP > PET), despite a much larger available surface area of smaller PET particles. The effect of a larger surface area was seen for PP MPs, where sPP bound more proteins than lPP (sPP > lPP). A higher intensity of protein bands in the SC and HC of lPP MPs was also found for BME sarcoplasmic proteins and meat allergens, showing their higher affinity for PP MPs. Protein adsorption onto PET is mainly based on the following: I) hydrophobic interactions between long aliphatic side chains and the PET surface, II) π–π interactions between aromatic amino acids and aromatic rings of terephthalate, and III) hydrogen bonds between amino acids and PET oxygens. In contrast, protein interactions with PP are exclusively hydrophobic. PP is the most hydrophobic plastic material with a LogP (SA)^−1^ value of about 25 × 10^−3^ Å^−2^, while PET is much less hydrophobic, with a LogP (SA)^−1^ around 8 × 10^−3^ Å^−2^ [47]. Proteins tend to adhere more strongly to nonpolar than to polar surfaces, as a nonpolar environment destabilizes proteins and thus facilitates a conformational rearrangement. This results in strong protein–surface hydrophobic interactions [48], which explains the observed higher affinity of trypsin to sPP MPs and BME proteins to lPP MPs.

Upon adsorption, PET MPs do not change trypsin’s tertiary structure in bulk solution, while both PP MPs induce only a slight loosening. However, trypsin’s secondary structure was more affected upon adsorption, particularly in the SC. All tested MPs slightly induced an increase in the ordered structure of trypsin in the SC and the slight rearrangement of its secondary structure, which was most pronounced for lPP MPs with an α to β transition. Upon adsorption of porcine trypsin to PS NPs, X. Li et al. [49] observed an α to β transition with a decrease in random content. Trypsin adsorption onto PVC NPs also resulted in a decrease in random content, but with a β to α transition [37]. Minor changes in the trypsin structure induced by MPs did not have negative effects on the specific activity in bulk and SC, but in contrast, exhibited slight positive effects, with the preservation of trypsin’s activity during prolonged incubation.

In the HC, specific activity was several times lower than the activity in the SC and bulk solution, most likely because trypsin bound in the HC contains a higher share of less active/inactive trypsin molecules. This is in accordance with trypsin binding with high affinity in HC, as was observed in adsorption isotherms, which is an irreversible process. However, even in the HC, where only a low trypsin fraction was found, there was residual trypsin activity of about 5–10%. As trypsin molecules in the HC make up 0.34–0.57% of total trypsin mass, it can be concluded that MPs have only negligible effects on trypsin activity. Although trypsin adsorption to 0.8 μm PVC NPs significantly affected trypsin’s secondary and tertiary structure, Liu et al. [37] observed only a slight decrease in its activity in bulk solution at an MP concentration of 0.08 mg/mL. This suggests that even when high-affinity trypsin adsorption onto MPs induces a significant structural change, trypsin activity is more or less preserved. In contrast to trypsin, our previous study [26] demonstrated that 10 μm PS MPs caused structural changes in pepsin in simulated gastric fluid, and at a concentration of 0.012 mg/mL, these MPs induced significant reduction in pepsin activity in bulk. Further, 10 μm-sized PS MPs at a concentration of 0.08 mg/mL reduced lipase activity by 23%, with changes in secondary structure [24]. This implies that when MPs are incubated with GIT enzymes, the preservation of enzyme activity depends on enzyme structure. Interestingly, even when MPs induced some structural change, this may or may not affect total enzyme activity. The current study demonstrates that trypsin activity seems to be less sensitive to MP-induced structural changes than pepsin and lipase.

There are three possible reasons why only slight effects of MPs on trypsin structure and activity were observed in this study. The first reason is the high trypsin concentration applied, relative to the MPs’ surface area. This could have prevented enzyme spreading upon adsorption, and thus caused more intensive conformational changes. It has been shown that only adsorption at low protein concentrations allows the high spreading and significant conformational changes of adsorbed proteins [50]. Secondly, in relation to the stability of proteins at solid–liquid interfaces and surface adsorption, proteins are classified as hard and soft, having high and low structural stability, respectively [51]. α-helices are more compressive than β-sheets and their unfolding requires less energy, which explains why soft proteins have a higher ratio of α-helix to β-sheet than hard proteins [52]. Similarly to α-chymotrypsin, categorized as a “hard” protein [53], trypsin is mainly composed of β-sheets, and thus has a low tendency for structural alterations upon surface adsorption. In contrast to trypsin, pancreatic lipase has a more complex structure, consisting of two distinct domains—the larger N-terminal domain with a typical α/β structure, and a smaller C-terminal domain of a β-sandwich type [54]. Indeed, studies have shown that PS MPs can decrease pancreatic lipase activity significantly, possibly due to structural changes [24,25]. Similarly, for human gastric lipase, which contains 41% α-helices and 14% β-sheets [55], it could be expected to show more pronounced activity changes upon adsorption to MP. Overall, it will contribute to the already observed impact of MP on lipid digestion, in addition to the impact of MP on the coalescence and aggregation of lipids, which also decreases the surface available for lipase action. This additional mechanism of MP interference with protein digestion is not to be expected. Thirdly, the Ca^2+^ extraction from trypsin by MP surfaces was completely prevented, as the uncharged surfaces of PP and PET MPs do not enable significant Ca^2+^ binding, and all experiments were performed in the presence of 0.3 or 0.6 mM Ca^2+^. In contrast, upon adsorption of α-lactalbumin, whose structure is also stabilized by Ca^2+^ ions, PS nanospheres induce its non-native conformation with a preserved secondary structure and loss of tertiary structure due to the strong binding of Ca^2+^ ions on negatively charged PS [56].

This study demonstrates that during digestion, both trypsin and BME proteins (including αGal-carrying allergens), as its substrates, bind to both PP and PET MPs. Further, they are found in the SC and HC of both MP types. However, BME proteins have a higher affinity for the more hydrophobic PP surface compared to PET. On the other hand, despite trypsin and BME proteins’ adsorption on the MP surface, and the fact that an extremely high MPs concentration (20 mg/mL MPs with 3 mg/mL meat proteins) was used, only slight effects were observed on the in vitro digestibility of BME proteins. Moreover, only the digestibility of BME proteins with masses of about 38 kDa was affected, leading to its slowing down. In addition, MPs did not affect the digestion of α-Gal meat allergens. In contrast, our previous study [26] demonstrated that the presence of 10 μm-sized polystyrene MPs has a negative effect on the pepsin digestion of cow’s milk proteins even at an MP concentration of 0.3 mg/mL MPs (with 7.5 mg/mL of milk proteins in the gastric digestion mixture).

There are two reasons why only slight effects of MPs on total BME protein and α-Gal-bearing meat allergens’ digestibility were observed. Firstly, it was demonstrated that the presence of MPs does not affect the trypsin activity, except for the low trypsin fraction in the HC. Moreover, during digestion, in the presence of an almost 50-fold higher mass of BME proteins competing with trypsin for adsorption on the plastic surface, the affected trypsin fraction in HC is even lower, thus an effect on the trypsin activity is even less likely. The second reason is that beef meat proteins are mainly (90%) digested during the gastric phase before reaching intestinal digestion [57]. Based on the α-Gal-binding properties, α-Gal allergens from BME are relatively resistant to gastric digestion, and completely resistant to subsequent intestinal digestion [58]. Hence, it is expected that in this study, BME proteins and allergens are only partially digested, and thus it is hard to observe a profound effect of MPs on meat protein digestion.

Although in our study trypsin activity, and consequentially BME digestion, was not directly impacted by the presence of MPs, it is still possible for MPs to have an indirect effect on food digestion and overall gut health. Studies on in vivo mouse models have found that the presence of MPs in the gut can lead to a plethora of changes, including decreased mucus production [59,60], damage to the intestinal barrier [59], inflammation [61] and changes in the gut microbiota [59,60,61]. The metabolic activity of the gut microbiota allows for the breakdown of complex indigestible carbohydrates and proteins [62], while the discontinuous nature of the mucus layer in the small intestine plays a role in the release of digestive enzymes in the brush border membrane of epithelial cells [63]. Additionally, polystyrene MPs have been found to increase hepatic total bile acids in mice [59], which could further impact the digestion of lipids. However, the concentrations of plastics used in these studies were above realistic exposure concentrations, as estimated by Mohamed Nor et al. [12].

Tan et al. [24] reported that PET MPs with comparable sizes and shapes to those used in this study decreased the in vitro intestinal digestion of lipids by about 10% at a concentration 250 times lower than that used in this study (80 µg/mL). This was due to the MPs-induced decrease in enzyme activity and the reduced availability of lipid droplets. In this study, during BME digestion, the tested MPs, even when present at a concentration of 20 mg/mL, did not influence enzyme activity, and only slightly influenced protein substrate susceptibility to trypsin action. However, it was demonstrated that the presence of MPs does not decrease the resistance of BME proteins, nor α-Gal allergens, to trypsin. Even in the presence of a 6.7-times higher mass of MPs than BME proteins, no increase in their digestibility was observed. Moreover, BME proteins and α-Gal allergens residing within the SC are prone to digestion at similar levels as proteins in solution, suggesting the same level of protein susceptibility to trypsin attack. These results suggest that, although the adsorption of BME proteins and α-Gal allergens onto MPs’ surfaces most likely changes their structure, at least slightly, this structural change is not sufficient to facilitate trypsin attack.

The SC and HC of BME proteins, as well as meat allergens, form quickly on both particle types within 5 min of contact with MPs (digestion time point 0 min). During digestion, though, both the SC and HC behave as a dynamic system, where, in addition to protein digestion by trypsin, the adsorption and desorption of proteins and their proteolytic products take place.

Moreover, this study demonstrated that both the SC and HC are rich in α-Gal allergens, behaving similarly to total BME proteins, with their dynamic adsorption and desorption.

In general, allergens are highly abundant proteins in foods, and their adsorption on the surfaces of MPs results in a corona enriched with allergenic proteins, as observed in this study. Upon their adsorption, changes in food allergen secondary and tertiary structures induced by MPs might lead to the exposure or disruption of conformational and/or linear epitopes, resulting in altered interactions with IgE and the affected food’s sensitizing and allergenic capacity [28]. Phue et al. [64] reported that milk allergens form a protein corona on SiO_2_ and TiO_2_ nanoparticles, and that particle-mediated alterations in the protein structure could enhance the allergenicity of milk proteins. It can therefore not be excluded that meat allergens residing in the SC, and particularly in the HC, or allergens desorbed into solution, may have altered IgE-binding properties and faithduring digestion.

This study demonstrated that neither MPs with a highly hydrophobic surface (PP) nor MPs with a moderately hydrophobic surface (PET) significantly affect trypsin activity. The estimated median steady state of MPs in the gut is about 300–500 particles/capita (0.8 to 1.6 ng/capita) [12]. Therefore, it is unlikely that the realistic exposure of humans to MPs in the intestine would have significant effects on BME proteins’ digestibility by trypsin. Although further investigations will reveal how the presence of MPs affects other intestinal proteases (e.g., chymotrypsin, carboxypeptidase, elastase, and particularly brush border peptidases), it is also unlikely that the presence of realistic MP amounts in the GIT would influence amino acids’ release and their overall bioavailability. However, due to the adsorption of BME allergens and their IgE-binding epitopes in the SC and HC, the presence of MPs in the GIT may affect their absorption, transportation, and subsequent presentation to the host immune system.

## 4. Materials and Methods

### 4.1. Materials

Trypsin from porcine pancreas (60 U/mg), cat. no. 37291.03, was purchased from SERVA Electrophoresis GmbH (Heidelberg, Germany). Fresh beef round was purchased from the local market. All reagents were of analytical grade. Ultrapure water (Barnstead Smart2Pure Water Purification System, Thermo Fischer Scientific, Waltham, MA, USA) was used for all experiments. 4-(2-aminoethyl)benzenesulfonyl fluoride hydrochloride (AEBSF), Nα-Benzoyl-DL-arginine 4-nitroanilide hydrochloride (BAPNA) and Nα-p-Tosyl-L-arginine methyl ester hydrochloride (TAME) were purchased from Sigma-Aldrich (St. Louis, MO, USA).

Microplastics (MPs) were produced at the University of Vienna, Department of Pharmaceutical Sciences, by milling and size fractionation as described in Section S3.1. Briefly, PP granules were cryomilled while keeping the temperature below the glass transition temperature (T_g_) of PP [65], fractionated by sieving through a stack of stainless-steel mesh sieves (63 µm, 180 µm, 500 µm), suspended in ethanol and filtered using a 10 µm nylon mesh filter to remove the fine particle fraction. PET particles were produced in an ultra-centrifugal mill following pre-cooling in liquid nitrogen, in order to keep the temperature below the T_g_ of PET [66], similarly to Ducoli et al. [67]. Size fractionation and the removal of fine particles were carried out by suspending the material in ethanol and filtering using a 0.8 µm nylon membrane. Before use, the ethanol was removed and the dry MP powder was stored at 4 °C. One size of PET and two sizes of PP were used (small—sPP and large—lPP). The particle size distributions of different MP fractions were determined by laser diffraction. The median size of PET was 55 µm (span: 22–106 µm), while the median sPP diameter was 117 µm (span: 58–238 µm), and the median diameter of the lPP was 326 µm (span: 187–494 µm) (Supplement Section S3.2, Figure S4, Table S2). The chemical composition (Section S2.2, Table S3, Figures S5–S7), size and shape (Figure S9A–C) of test materials were characterized using microFTIR. The identity of each test material was confirmed with a 90.21% match to PET reference spectra, and 86.55% and 89.12% matches to PP reference spectra for sPP and lPP, respectively. Sizes were in accordance with the sizes obtained through laser diffraction, and the particles showed an irregular shape.

### 4.2. Trypsin Binding to MPs

Unless otherwise stated, all incubations of trypsin with MPs were performed at room temperature in simulated intestinal fluid (SIF) buffer, pH 7 (see Section S4, Table S4) [68], in glass vials. Incubation mixtures were constantly mixed by Bio RS-24 mini rotator (BioSan, Latvia). Trypsin adsorption could only be measured with PET MPs since PP MPs float in aqueous solutions due to their low density (0.9 g/cm^3^), and a reliable system equilibration necessary for adsorption analysis could not be obtained. The characterization of trypsin binding (affinity and capacity) to PET MPs was performed first by the determination of equilibration time, whereby the processes of adsorption and desorption are at equilibrium. This was done by incubating 20 mg of tested MPs with 1 mg of trypsin in 1 mL of SIF. After 1, 2, 3 and 4 h of incubation, MPs were separated from the trypsin solution by centrifugation (11,340× *g*, MiniSpin centrifuge, Eppendorf, Germany) and filtration (0.22 µm), followed by measurement of the absorbance in the supernatant at 280 nm using the NanoDrop 2000c (Thermo Fisher Scientific, USA). The concentration of trypsin was calculated using the extinction coefficient for trypsin of ε_1%_ = 15 at 280 nm [69].

After the equilibration time was determined (1 h, Section S1.1, Figure S1), the following experiment for the calculation of binding parameters was performed. Trypsin concentrations of 0.125, 0.25, 0.5, 0.75 and 1 mg/mL were tested in the presence of 20 mg of PET MPs in a total volume of 1 mL SIF. At the equilibration point, supernatants were separated from MPs and trypsin concentrations in the supernatant were determined by measuring the absorbance at 280 nm. Binding experiments were performed in triplicates.

Binding parameters were obtained by fitting the obtained data into several binding isotherms, including Langmuir, Freundlich, Redlich–Peterson (RP) and Guggenheim–Anderson–de Boer (GAB) (Section S1.2, Figure S2). Fitting was performed with the OriginPro 8.5.1 software and non-linear regression analysis was used for the calculation of binding parameters.

### 4.3. Formation of Soft and Hard Corona on the Surface of Tested MPs

To test whether trypsin can form soft and hard coronae on the surface of MPs, 1 mL of trypsin at a final concentration of 1 mg/mL in SIF was incubated with 20 mg of each MPs type. Soft and hard corona samples were obtained following a published procedure [70]. For soft corona samples, pelleted MPs were washed three times with 200 µL of ultrapure water. Each washing step consisted of water addition, vortexing for 30 s, incubation in water for 5 min, and the subsequent removal of MPs by centrifugation. To obtain trypsin from the hard corona of washed MPs, 200 µL of sodium dodecyl–sulfate polyacrylamide gel electrophoresis (SDS PAGE) sample buffers (both reducing and non-reducing) were added, and the samples were boiled for 10 min at 95 °C. All the samples were resolved via SDS PAGE in reducing and non-reducing conditions on 14% hand-cast gels according to the method of Laemmli [71], whereby 30 µL was applied per well. For the detection of protein bands, silver staining was performed using the protocol of Chevallet et al. [72].

### 4.4. Influence of MPs on the Structure of Trypsin Analyzed by Circular Dichroism (CD) Spectrometry

For structural analysis of trypsin upon interaction with different types of MPs, trypsin in bulk solution and the soft corona was analyzed by CD spectrometry using a Jasco J-815 CD spectropolarimeter (JASCO, Tokyo, Japan). Briefly, the trypsin solution in SIF (1 mg/mL) was incubated at room temperature with different MPs (20 mg/mL) for 0, 1, 2 and 4 h, under constant mixing. The supernatant was removed from MPs by centrifugation (1000× *g*, 2 min) and filtration (PVDF, 0.22 µm) and CD spectra of the supernatant (bulk trypsin solution) were recorded. The remaining MPs were washed once with 5 mM sodium phosphate buffer at pH 7 instead of water to obtain the soft corona trypsin samples. To obtain bulk samples designated as 0 h after the addition of MPs to trypsin solution, the sample was vortexed and immediately centrifugated, the supernatant was separated, and CD spectra were immediately recorded. Similarly, for SC samples from pelleted MPs designated as 0 h, SCs were immediately isolated, and CD spectra were recorded. Near UV CD spectra were recorded in the range of 260 to 320 nm, using a cuvette with a 10 mm path length, while far UV CD spectra were recorded in the range of 190 to 260 nm in a cuvette with 1 mm path length under thermostatic conditions (20 °C). Spectra were recorded in 0.1 nm steps with a 50 nm/min scan speed. Each sample was recorded four times. Both the near and far UV CD spectra of SIF and SIF diluted seven times with phosphate buffer were recorded and subtracted from appropriate sample spectra. The results have been normalized and are presented as molar ellipticity. For the determination of the amount of secondary structural motifs, far UV CD spectra were converted to molar ellipticity per residue using the following equation:

(1) $$\left[\theta \right]=\frac{\theta \times Mr}{10\times r\times l\times [T]}$$

where [θ] is the molar ellipticity per residue in (deg × cm^2^)/dmol, θ is ellipticity in mdeg, Mr is the molar mass of trypsin, r is the number of amino acids in trypsin (223), l is the path length in cm and [T] is the concentration of trypsin used in mg/mL. For the calculation of secondary structure motifs, the obtained results were further analyzed in the CDpro software package, using the CONTIN algorithm. The SP29 database was used for the analysis.

### 4.5. Influence of MPs on Trypsin Activity

The influence of MPs on the activity of trypsin in bulk and MPs soft and hard coronae was assessed at room temperature using BAPNA as a substrate at a final concentration of 1 mM. Trypsin-specific activity (U/mg) was calculated using the extinction coefficient Ɛ405 for p-nitroanilide nm 9900 M^−1^ cm^−1^ [73], where one unit of trypsin per mg (U/mg) is defined as 1 μmol BAPNA hydrolyzed per min per mg of enzyme.

#### 4.5.1. Trypsin Activity in Bulk and MPs Soft Corona

A trypsin solution (1 mg/mL) prepared in 1 mL of SIF was incubated with or without MPs (20 mg) for 0, 1, 2, and 4 h. At the mentioned time points, bulk and soft corona trypsin samples were obtained as described in Section 4.3, but using SIF for all MPs rinsing steps. The reaction mixture consisted of 5 µL of bulk or soft corona solution, 193 µL of SIF, and 2 µL of BAPNA (100 mM) in DMSO in a 96-well microplate. The reaction mixture was immediately mixed and absorbance at 405 nm was monitored for 5 min. Each sample was prepared as a biological duplicate, and activity was measured twice in each. As a negative control, BAPNA was incubated in SIF for the same duration, while the activity of trypsin without the presence of MPs was monitored through time as a positive control.

#### 4.5.2. Activity of Trypsin in the Hard Corona

MPs (40 mg) were incubated with trypsin (1 mg/mL) in 2 mL of SIF solution for 1 h at room temperature under the conditions described in Section 4.5.1. Upon removing bulk trypsin by centrifugation, MPs were washed with SIF solution to obtain soft corona trypsin as described in Section 4.3. The MP fraction was then mixed with 300 µL of fresh SIF solution with BAPNA substrate in a final concentration of 1 mM. The reaction mixture was incubated for 10 min with constant rotation and centrifuged for 2 min at 1000× *g*, the supernatant was collected, and the absorbance of the supernatant was measured at 405 nm 14 min after BAPNA was added in a cuvette with 10 mm path length. As a negative control, BAPNA was incubated in SIF for the same duration. For the semi-quantitative determination of the amount of trypsin in the MP hard corona, 75 µL of reducing 5x Laemmli buffer was added into the remaining MPs pellet together with the reaction mixture. Samples were prepared for resolving via SDS PAGE on 15% polyacrylamide gels under reducing conditions, where 30 µL was transferred into each well. The semi-quantification of trypsin in HC was done relative to a trypsin preparation applied in the same gel. Gels were analyzed after silver staining using ImageJ 1.54 software and the integrated optical density of each band was calculated.

### 4.6. Influence of MPs on In Vitro Digestion of Meat Extract Proteins with Trypsin

#### In Vitro Digestion of Meat Extract Proteins with Trypsin

Beef meat extract (BME) was prepared by meat extraction with simulated salivary fluid (SSF) at pH 7 [68], according to the procedure described in Section S5, using a previously described protocol [74]. Briefly, beef meat was homogenized in SSF, and extracted for 1 h at 4 °C, followed by centrifugation and filtration. The BME obtained by extraction with low-ionic-strength buffer contained mostly sarcoplasmic meat proteins, and their profiles are presented in Figure S10. Before the digestion experiment, BME was centrifuged for 5 min at 11,340× *g*. A different batch of PET but with similar characteristics was used for this experiment (see PET 2 in Supplementary Section S3.2, Figures S4, S8 and S9, Tables S2 and S3). In vitro meat protein digestion with trypsin was performed in 0.5× simulated intestinal fluid (SIF), pH 7 [68], in the presence of 20 mg/mL PET and lPP. The final concentrations of trypsin and BME proteins in the digestion mixture were 0.063 mg/mL, with a total activity of 6.13 U/mL, and 3 mg/mL, respectively. Trypsin activity was determined according to Hummel [75], whereby one unit hydrolyses 1 µmol of p-toluene-sulfonyl-L-arginine methyl ester (TAME) per minute at 25 °C and at pH 8.1, as recommended in Brodkorb et al. [68]. The ratio (*w*/*w*) of MPs:trypsin:meat proteins was 317.5:1:47.4. Controls were prepared in the same way, excluding only the MPs. Samples were vortexed and placed in a rotator at 37 °C. Digestion was performed for 0, 10, 60 and 120 min. To stop the digestion, 100 mM AEBSF with a final concentration of 4.76 mM was added to the samples and thoroughly vortexed. Samples were centrifuged for 5 min at 6700× *g* to remove bulk solution. Soft coronae samples were obtained as described in Section 4.2, using SIF instead of water. For the 0 min time point, AEBSF was added before the trypsin and the samples were centrifuged immediately. To obtain hard coronae, reducing SDS PAGE buffer was added to the precipitated MPs, and incubated for 10 min at 95 °C. Bulk samples were analyzed on 12% polyacrylamide gels, while SC and HC samples were analyzed on 14% gels. For the SDS PAGE analysis of SC, aliquots of the first washings were applied. Immunoblots for the detection of α-Gal epitope in digested beef meat proteins were performed using commercial human monoclonal IgM antibodies to α-Gal (Abcam, Cambridge, UK), and the process is described in detail in the Supplementary Material (Section S6).

### 4.7. Statistics and Graph Generation

The results are expressed as mean ± standard deviation (S.D.) where applicable. The statistical significance of the obtained differences between samples was tested using one-way ANOVA with Tukey’s multiple comparison test. All statistical analyses and graphical representations of data were performed using GraphPad Prism 8.0.2. *p* values less than 0.05 were considered as statistically significant.

## 5. Conclusions

Human exposure to MPs through various routes, particularly ingestion, raises concerns about their potential interference with food digestion. This is particularly significant given that the intestinal tract is the site of sensitization to food allergens, including the emerging AGS. This study demonstrated the adsorption of trypsin on both PP and PET MPs, but resulting only in slight structural changes, and negligibly affecting trypsin specific activity. During trypsin digestion, the corona on PP and PET MPs is composed of BME sarcoplasmic proteins and α-Gal-carrying proteins. The presence of MPs slightly slowed down the digestion of BME proteins with a mass of about 38 kDa, and no increase in digestibility was observed. Further, MPs did not show an effect on the digestibility of α-Gal allergens. Although found in the SC and HC of both MP types, trypsin and BME proteins, including α-Gal allergens, have a higher affinity to MPs with a highly hydrophobic surface (PP) than to those with a moderately hydrophobic surface (PET). This effect was observed despite the much larger available surface area of PET compared to PP MPs. However, the presence of α-Gal allergens in the SC and HC of MPs and their dynamic compositions during digestion due to adsorption and desorption indicate the possibility of their altered IgE binding properties as induced by MPs, which should be further investigated. In general, this study implies that it is unlikely that the real-life exposure to MPs would have any significant effect on BME’s digestibility by trypsin, but the presence of MPs in the GIT and the presence of BME sarcoplasmic proteins and α-Gal-carrying proteins in hard corona during trypsin digestion may influence the potential of allergens for sensitization and allergic reactions.

## Figures and Tables

**Table 1:** Adsorption parameters of trypsin on PET MPs obtained by non-linear regression analysis using different binding isotherms.

Isotherm Model	Parameters of Isotherms PET	
**Langmuir**	K_L_ (mL/mg)	10.15
	Q_L_ (mg/g)	4.16
	R_L_	0.078
	R^2^	0.9623
**Freundlich**	K_F_ ((mg/g) * (mL/mg)^1/n^)	4.08
	n	3.7
	R^2^	0.8267
**Redlich–eterson**	K_R_ (mL/mg)	28.2
	α (mL/mg)	1.2
	β	1.26
	R^2^	0.9970
**Guggenheim–Anderson–de Boer**	Q_G_ (mg/g)	7.15
	K_hard G_ (mL/mg)	4.5
	K_soft G_ (mL/mg)	−0.56
	R^2^	0.9945

## Data Availability

The original data presented in the study are openly available in the Faculty of Chemistry Repository—Cherry, at https://cherry.chem.bg.ac.rs/handle/123456789/7064 (accessed on 16 June 2025).

## Supplementary Materials

The following supporting information can be downloaded at: https://www.mdpi.com/article/10.3390/ijms26135974/s1.

## References

[1] Koelmans A.A., Redondo-Hasselerharm P.E., Nor N.H.M., de Ruijter V.N., Mintenig S.M., Kooi M. (2022). Risk Assessment of Microplastic Particles. Nat. Rev. Mater..

[2] Di Fiore C., Carriera F., Russo M.V., Avino P. (2023). Are Microplastics a Macro Issue? A Review on the Sources of Contamination, Analytical Challenges and Impact on Human Health of Microplastics in Food. Foods.

[3] Giri S., Lamichhane G., Khadka D., Devkota H.P. (2024). Microplastics Contamination in Food Products: Occurrence, Analytical Techniques and Potential Impacts on Human Health. Curr. Res. Biotechnol..

[4] Kumar V., Sharma N., Umesh M., Gupta P., Sharma P., Basheer T., Huligowda L.K.D., Thomas J., Bhagat S.K., Pasrija R. (2024). Microplastics in Food: Occurrence, Toxicity, Green Analytical Detection Methods and Future Challenges. Green Anal. Chem..

[5] Udovicki B., Andjelkovic M., Cirkovic-Velickovic T., Rajkovic A. (2022). Microplastics in Food: Scoping Review on Health Effects, Occurrence, and Human Exposure. Int. J. Food Contam..

[6] Vitali C., Peters R.J.B., Janssen H.-G., Nielen M.W.F. (2023). Microplastics and Nanoplastics in Food, Water, and Beverages; Part I. Occurrence. TrAC Trends Anal. Chem..

[7] Huerta Lwanga E., Mendoza Vega J., Ku Quej V., Chi J.D.L.A., Sanchez del Cid L., Chi C., Escalona Segura G., Gertsen H., Salánki T., van der Ploeg M. (2017). Field Evidence for Transfer of Plastic Debris along a Terrestrial Food Chain. Sci. Rep..

[8] Habib R.Z., Poulose V., Alsaidi R., al Kendi R., Iftikhar S.H., Mourad A.-H.I., Kittaneh W.F., Thiemann T. (2022). Plastic Cutting Boards as a Source of Microplastics in Meat. Food Addit. Contam..

[9] Kaseke T., Lujic T., Cirkovic Velickovic T. (2023). Nano- and Microplastics Migration from Plastic Food Packaging into Dairy Products: Impact on Nutrient Digestion, Absorption, and Metabolism. Foods.

[10] Kedzierski M., Lechat B., Sire O., Le Maguer G., Le Tilly V., Bruzaud S. (2020). Microplastic Contamination of Packaged Meat: Occurrence and Associated Risks. Food Packag. Shelf Life.

[11] Li D., Shi Y., Yang L., Xiao L., Kehoe D.K., Gun’ko Y.K., Boland J.J., Wang J.J. (2020). Microplastic Release from the Degradation of Polypropylene Feeding Bottles during Infant Formula Preparation. Nat. Food.

[12] Mohamed Nor N.H., Kooi M., Diepens N.J., Koelmans A.A. (2021). Lifetime Accumulation of Microplastic in Children and Adults. Environ. Sci. Technol..

[13] Pletz M. (2022). Ingested Microplastics: Do Humans Eat One Credit Card per Week?. J. Hazard. Mater. Lett..

[14] Schwabl P., Köppel S., Königshofer P., Bucsics T., Trauner M., Reiberger T., Liebmann B. (2019). Detection of Various Microplastics in Human Stool: A Prospective Case Series. Ann. Intern. Med..

[15] Yan Z., Liu Y., Zhang T., Zhang F., Ren H., Zhang Y. (2022). Analysis of Microplastics in Human Feces Reveals a Correlation between Fecal Microplastics and Inflammatory Bowel Disease Status. Environ. Sci. Technol..

[16] Zhang N., Li Y.B., He H.R., Zhang J.F., Ma G.S. (2021). You Are What You Eat: Microplastics in the Feces of Young Men Living in Beijing. Sci. Total Environ..

[17] Ke D., Zheng J., Liu X., Xu X., Zhao L., Gu Y., Yang R., Liu S., Yang S., Du J. (2023). Occurrence of Microplastics and Disturbance of Gut Microbiota: A Pilot Study of Preschool Children in Xiamen, China. eBioMedicine.

[18] Zhang J., Wang L., Trasande L., Kannan K. (2021). Occurrence of Polyethylene Terephthalate and Polycarbonate Microplastics in Infant and Adult Feces. Environ. Sci. Technol. Lett..

[19] Özsoy S., Gündogdu S., Sezigen S., Tasalp E., Ikiz D.A., Kideys A.E. (2024). Presence of Microplastics in Human Stomachs. Forensic Sci. Int..

[20] Cetin M., Demirkaya Miloglu F., Kilic Baygutalp N., Ceylan O., Yildirim S., Eser G., Gul H.İ. (2023). Higher Number of Microplastics in Tumoral Colon Tissues from Patients with Colorectal Adenocarcinoma. Environ. Chem. Lett..

[21] Ibrahim Y.S., Tuan Anuar S., Azmi A.A., Wan Mohd Khalik W.M.A., Lehata S., Hamzah S.R., Ismail D., Ma Z.F., Dzulkarnaen A., Zakaria Z. (2021). Detection of Microplastics in Human Colectomy Specimens. JGH Open.

[22] Yin K., Wang Y., Zhao H., Wang D., Guo M., Mu M., Liu Y., Nie X., Li B., Li J. (2021). A Comparative Review of Microplastics and Nanoplastics: Toxicity Hazards on Digestive, Reproductive and Nervous System. Sci. Total Environ..

[23] Monopoli M.P., Åberg C., Salvati A., Dawson K.A. (2012). Biomolecular Coronas Provide the Biological Identity of Nanosized Materials. Nat. Nanotech..

[24] Tan H., Yue T., Xu Y., Zhao J., Xing B. (2020). Microplastics Reduce Lipid Digestion in Simulated Human Gastrointestinal System. Environ. Sci. Technol..

[25] Zhu H., Wu P., Hu Z., Chen H., Wang N., Chen X.D. (2025). Unraveling the Impact of Polystyrene Microplastics with Varying Particle Sizes and Concentrations on Lipid In Vitro Digestion and Ex Vivo Absorption. J. Hazard. Mater..

[26] de Guzman M.K., Stanic-Vucinic D., Gligorijevic N., Wimmer L., Gasparyan M., Lujic T., Vasovic T., Dailey L.A., Van Haute S., Cirkovic Velickovic T. (2023). Small Polystyrene Microplastics Interfere with the Breakdown of Milk Proteins during Static In Vitro Simulated Human Gastric Digestion. Environ. Pollut..

[27] Pekar J., Ret D., Untersmayr E. (2018). Stability of Allergens. Mol. Immunol..

[28] Shi Q., Wang Z., Wu Y., Chen H., Gao J. (2024). Oral Exposure to Nano- and Microplastics: Potential Effects in Food Allergies?. Allergy Med..

[29] Hou G., Hu W., Zhao J., Lu J., Zhang W., Liu X., Lu S., Shinichi Y., Ebere E.C., Wang Q. (2025). Studies on Adsorption and Synergistic Biological Effects Induced by Microplastic Particles and the *Platanus* Pollen Allergenic Protein 3 (Pla a3). Environ. Pollut..

[30] DeLoid G.M., Cao X., Coreas R., Bitounis D., Singh D., Zhong W., Demokritou P. (2022). Incineration-Generated Polyethylene Micro-Nanoplastics Increase Triglyceride Lipolysis and Absorption in an In Vitro Small Intestinal Epithelium Model. Environ. Sci. Technol..

[31] Perusko M., Grundström J., Eldh M., Hamsten C., Apostolovic D., van Hage M. (2024). The α-Gal Epitope—The Cause of a Global Allergic Disease. Front. Immunol..

[32] Huang J.-N., Wen B., Zhu J.-G., Zhang Y.-S., Gao J.-Z., Chen Z.-Z. (2020). Exposure to Microplastics Impairs Digestive Performance, Stimulates Immune Response and Induces Microbiota Dysbiosis in the Gut of Juvenile Guppy (*Poecilia reticulata*). Sci. Total. Environ..

[33] Wang X., Huang W., Wei S., Shang Y., Gu H., Wu F., Lan Z., Hu M., Shi H., Wang Y. (2020). Microplastics Impair Digestive Performance but Show Little Effects on Antioxidant Activity in Mussels under Low pH Conditions. Environ. Pollut..

[34] Xiao K., Song L., Li Y., Li C., Zhang S. (2023). Dietary Intake of Microplastics Impairs Digestive Performance, Induces Hepatic Dysfunction, and Shortens Lifespan in the Annual Fish *Nothobranchius guentheri*. Biogerontology.

[35] Frank Y.A., Interesova E.A., Solovyev M.M., Xu J., Vorobiev D.S. (2023). Effect of Microplastics on the Activity of Digestive and Oxidative-Stress-Related Enzymes in Peled Whitefish (*Coregonus peled* Gmelin) Larvae. Int. J. Mol. Sci..

[36] Romano N., Ashikin M., Teh J.C., Syukri F., Karami A. (2018). Effects of Pristine Polyvinyl Chloride Fragments on Whole Body Histology and Protease Activity in Silver Barb *Barbodes gonionotus* Fry. Environ. Pollut..

[37] Liu G., Jiang Q., Qin L., Zeng Z., Zhang P., Feng B., Liu X., Qing Z., Qing T. (2024). The Influence of Digestive Tract Protein on Cytotoxicity of Polyvinyl Chloride Microplastics. Sci. Total Environ..

[38] Santos A.M.C., de Oliveira J.S., Bittar E.R., da Silva A.L., Guia M.L.D.M., Bemquerer M.P., Santoro M.M. (2008). Improved Purification Process of β- and α-Trypsin Isoforms by Ion-Exchange Chromatography. Braz. Arch. Biol. Technol..

[39] Perutka Z., Šebela M. (2018). Pseudotrypsin: A Little-Known Trypsin Proteoform. Molecules.

[40] Rabe M., Verdes D., Seeger S. (2011). Understanding Protein Adsorption Phenomena at Solid Surfaces. Adv. Colloid Interface Sci..

[41] Yue Y., Tu Q., Guo Y., Wang Y., Xu Y., Zhang Y., Liu J. (2022). Comparison of the Interactions of Fanetizole with Pepsin and Trypsin: Spectroscopic and Molecular Docking Approach. J. Mol. Liq..

[42] Gilliland G.L., Teplyakov A., Scott R.A. (2011). Structural Calcium (Trypsin, Subtilisin). Encyclopedia of Inorganic and Bioinorganic Chemistry.

[43] Koutsopoulos S., Patzsch K., Bosker W.T.E., Norde W. (2007). Adsorption of Trypsin on Hydrophilic and Hydrophobic Surfaces. Langmuir.

[44] Santos A.M.C., Santana M.A., Gomide F.T.F., Miranda A.A.C., Oliveira J.S., Vilas Boas F.A.S., Vasconcelos A.B., Bemquerer M.P., Santoro M.M. (2008). Physical-Chemical Characterization and Stability Study of Alpha-Trypsin at pH 3.0 by Differential Scanning Calorimetry. Int. J. Biol. Macromol..

[45] Covello C., Di Vincenzo F., Cammarota G., Pizzoferrato M. (2024). Micro(Nano)Plastics and Their Potential Impact on Human Gut Health: A Narrative Review. Curr. Issues Mol. Biol..

[46] Jones L.R., Wright S.J., Gant T.W. (2023). A Critical Review of Microplastics Toxicity and Potential Adverse Outcome Pathway in Human Gastrointestinal Tract Following Oral Exposure. Toxicol. Lett..

[47] Min K., Cuiffi J.D., Mathers R.T. (2020). Ranking Environmental Degradation Trends of Plastic Marine Debris Based on Physical Properties and Molecular Structure. Nat. Commun..

[48] Anand G., Sharma S., Dutta A.K., Kumar S.K., Belfort G. (2010). Conformational Transitions of Adsorbed Proteins on Surfaces of Varying Polarity. Langmuir.

[49] Li X., Wang Y., Hu S., Zong W., Liu R. (2024). New Mechanistic Insights of Nanoplastics Synergistic Cadmium Induced Overactivation of Trypsin: Joint Analysis from Protein Multi-Level Conformational Changes and Computational Modeling. J. Hazard. Mater..

[50] Cordeiro A.L., Rückel M., Bartels F., Maitz M.F., Renner L.D., Werner C. (2019). Protein Adsorption Dynamics to Polymer Surfaces Revisited-A Multisystems Approach. Biointerphases.

[51] Arai T., Norde W. (1990). The Behavior of Some Model Proteins at Solid-Liquid Interfaces 1. Adsorption from Single Protein Solutions. Colloids Surf..

[52] Coglitore D., Janot J.-M., Balme S. (2019). Protein at Liquid Solid Interfaces: Toward a New Paradigm to Change the Approach to Design Hybrid Protein/Solid-State Materials. Adv. Colloid. Interface Sci..

[53] Baron M.H., Revault M., Servagent-Noinville S., Abadie J., Quiquampoix H. (1999). Chymotrypsin Adsorption on Montmorillonite: Enzymatic Activity and Kinetic FTIR Structural Analysis. J. Colloid Interface Sci..

[54] Winkler F.K., D’Arcy A., Hunziker W. (1990). Structure of Human Pancreatic Lipase. Nature.

[55] Roussel A., Canaan S., Egloff M.-P., Rivière M., Dupuis L., Verger R., Cambillau C. (1999). Crystal Structure of Human Gastric Lipase and Model of Lysosomal Acid Lipase, Two Lipolytic Enzymes of Medical Interest. J. Biol. Chem..

[56] Engel M.F.M., van Mierlo C.P.M., Visser A.J.W.G. (2002). Kinetic and Structural Characterization of Adsorption-Induced Unfolding of Bovine α-Lactalbumin. J. Biol. Chem..

[57] Qiu Z., Shi Y., Zheng Y., Shi W., Zhang L., Yin M., Wang X. (2025). Comparison of in Vitro Digestive Characteristics of Proteins from Different Sources in Simulated Elderly Gastrointestinal Conditions. Food Chem..

[58] Benedé S., López-Fandiño R., Molina E. (2024). Residual α-Gal in Digested Beef, Pork and Lamb Meat Submitted to Different Cooking Methods. LWT.

[59] Jin Y., Lu L., Tu W., Luo T., Fu Z. (2019). Impacts of Polystyrene Microplastic on the Gut Barrier, Microbiota and Metabolism of Mice. Sci. Total Environ..

[60] Lu L., Wan Z., Luo T., Fu Z., Jin Y. (2018). Polystyrene Microplastics Induce Gut Microbiota Dysbiosis and Hepatic Lipid Metabolism Disorder in Mice. Sci. Total Environ..

[61] Li B., Ding Y., Cheng X., Sheng D., Xu Z., Rong Q., Wu Y., Zhao H., Ji X., Zhang Y. (2020). Polyethylene Microplastics Affect the Distribution of Gut Microbiota and Inflammation Development in Mice. Chemosphere.

[62] Schippa S., Conte M.P. (2014). Dysbiotic Events in Gut Microbiota: Impact on Human Health. Nutrients.

[63] Herath M., Hosie S., Bornstein J.C., Franks A.E., Hill-Yardin E.L. (2020). The Role of the Gastrointestinal Mucus System in Intestinal Homeostasis: Implications for Neurological Disorders. Front. Cell Infect. Microbiol..

[64] Phue W.H., Xu K., George S. (2022). Inorganic Food Additive Nanomaterials Alter the Allergenicity of Milk Proteins. Food Chem. Toxicol..

[65] Maddah H.A. (2016). Polypropylene as a Promising Plastic: A Review. Am. J. Polym. Sci..

[66] Thomsen T.B., Hunt C.J., Meyer A.S. (2022). Influence of Substrate Crystallinity and Glass Transition Temperature on Enzymatic Degradation of Polyethylene Terephthalate (PET). N. Biotechnol..

[67] Ducoli S., Federici S., Nicsanu R., Zendrini A., Marchesi C., Paolini L., Radeghieri A., Bergese P., Depero L.E. (2022). A Different Protein Corona Cloaks “True-to-Life” Nanoplastics with Respect to Synthetic Polystyrene Nanobeads. Environ. Sci. Nano.

[68] Brodkorb A., Egger L., Alminger M., Alvito P., Assunção R., Ballance S., Bohn T., Bourlieu-Lacanal C., Boutrou R., Carrière F. (2019). INFOGEST Static in Vitro Simulation of Gastrointestinal Food Digestion. Nat. Protoc..

[69] Buck F.F., Vithayathil A.J., Bier M., Nord F.F. (1962). On the Mechanism of Enzyme Action. 73. Studies on Trypsins from Beef, Sheep and Pig Pancreas. Arch. Biochem. Biophys..

[70] Magrì D., Sánchez-Moreno P., Caputo G., Gatto F., Veronesi M., Bardi G., Catelani T., Guarnieri D., Athanassiou A., Pompa P.P. (2018). Laser Ablation as a Versatile Tool To Mimic Polyethylene Terephthalate Nanoplastic Pollutants: Characterization and Toxicology Assessment. ACS Nano.

[71] Laemmli U.K. (1970). Cleavage of Structural Proteins during the Assembly of the Head of Bacteriophage T4. Nature.

[72] Chevallet M., Luche S., Rabilloud T. (2006). Silver Staining of Proteins in Polyacrylamide Gels. Nat. Protoc..

[73] Herrera-Camacho I., Rosas-Murrieta N., Rawlings N.D., Salvesen G. (2013). Leucyl Aminopeptidase *yspII* (Yeast). Handbook of Proteolytic Enzymes.

[74] Apostolovic D., Tran T.A.T., Hamsten C., Starkhammar M., Cirkovic Velickovic T., van Hage M. (2014). Immunoproteomics of Processed Beef Proteins Reveal Novel Galactose-α-1,3-Galactose-Containing Allergens. Allergy.

[75] Hummel B.C. (1959). A Modified Spectrophotometric Determination of Chymotrypsin, Trypsin, and Thrombin. Can. J. Biochem. Physiol..

