# Supplementary material for: Effects of Polypropylene and Polyethylene Terephthalate Microplastics on Trypsin Structure and Function
Source: Int J Mol Sci. 2025 Jun 21;26(13):5974. doi: 10.3390/ijms26135974 (PMC12249632; doi:10.3390/ijms26135974)
Supplement: Supplementary file 1 [file ijms-26-05974-s001.zip › ijms-3687129-supplementary.pdf]

Supplementary material

Effects of polypropylene and polyethylene terephthalate microplastics on trypsin structure and function

Tamara Lujic[1], Nikola Gligorijevic[2], Dragana Stanic-Vučinic[1], Maja Krstic Ristivojevic[1], Tamara Mutic[1], Lukas Wimmer[3,4], Lea Ann Dailey[3], Tanja Cirkovic Velickovic[1,5]

[1] University of Belgrade – Faculty of Chemistry, Studentski trg 12-16, 11000, Belgrade, Serbia

[2] University of Belgrade – Institute of Chemistry, Technology and Metallurgy, National Institute of the Republic of Serbia, Njegoseva 12, 11000, Belgrade, Serbia

[3] University of Vienna, Department of Pharmaceutical Sciences, Josef-Holaubek-Platz 2, 1090, Vienna, Austria

[4] University of Vienna, Doctoral School of Pharmaceutical, Nutritional and Sport Sciences, Josef-Holaubek-Platz 2, 1090, Vienna, Austria

[5] Serbian Academy of Sciences and Arts, Knez Mihailova 35, 11000, Belgrade, Serbia

Section S1. Determination of binding parameters of trypsin to MPs

S1.1. Determination of the equilibrium time for trypsin binding to PET and PP

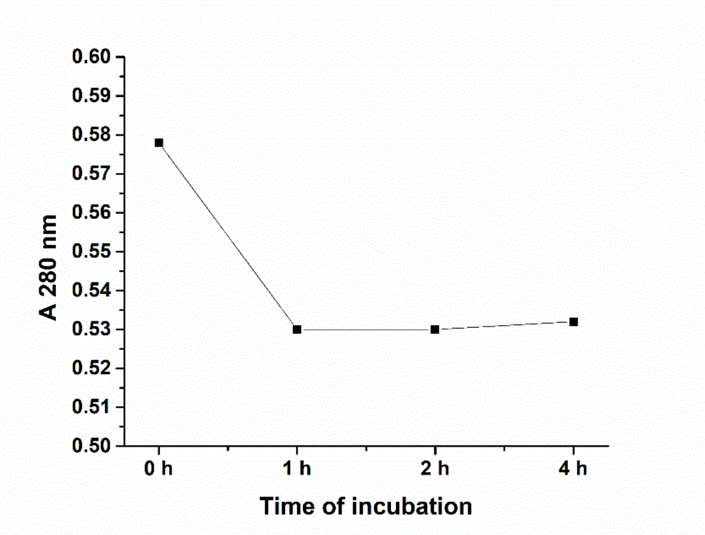

Figure S1. Determination of equilibrium time for trypsin binding to PET MPs.

S1.2. Calculation of binding parameters of trypsin to MPs

The following equation was used for the Langmuir adsorption isotherm:

$$Qe = \frac{Q_L \, x \, K_L \, x \, Ce}{1 + K_L \, x \, Ce}, \qquad (1)$$

Where Qe (mg/g) is the mass of adsorbed trypsin per gram of MPs at equilibrium, Ce (mg/L) is the concentration of unbound trypsin in solution at the equilibration point, Q_L (mg/g) is the maximum amount of adsorbed trypsin per mass unit of tested MPs and K_L (mL/mg) is the Langmuir affinity constant.

Using the Langmuir model, the separation factor or equilibrium parameter (R_L) is calculated by applying the following equation:

$$R_L = \frac{1}{1 + K_L \, x \, C_0}, \qquad (2)$$

where K_L represents the Langmuir constant and C_0 is the highest initial concentration of trypsin used.

The following equation was used for Freundlich adsorption isotherm:

$$Qe = K_F \, x \, Ce^{\frac{1}{n}}, \qquad (3)$$

where n is a dimensionless reaction order representing the measurement of heterogeneity and K_F ((mg/g)*(mL/mg)$^{1/n}$) is the Freundlich constant.

The following equation was used for RP adsorption isotherm:

$$Qe = \frac{K_R \, x \, Ce}{1 + \alpha Ce\beta'}, \qquad (4)$$

where K_R (mL/mg) represents Redlich–Peterson constant, α represents a constant in mL/mg and β is an exponent that usually falls in the range from 0 to 1. If β is closer to 1, the model approaches the Langmuir isotherm and if it is closer to 0, it approaches the Freundlich isotherm. For GAB adsorption isotherm, the equation used was as follows:

$$Qe = \frac{Q_G \, x \, K_{hard\,G} \, x \, Ce}{(1 - K_{soft\,G} \, x \, Ce)(1 + K_{hard\,G} - K_{soft\,G} \, x \, Ce)}, \quad (5)$$

where Q_G (mg/g) is the surface concentration of strongly adsorbed trypsin, $K_{hard\,G}$ (mL/mg) is the hard corona adsorption constant, and $K_{soft\,G}$ is the soft corona adsorption constant.

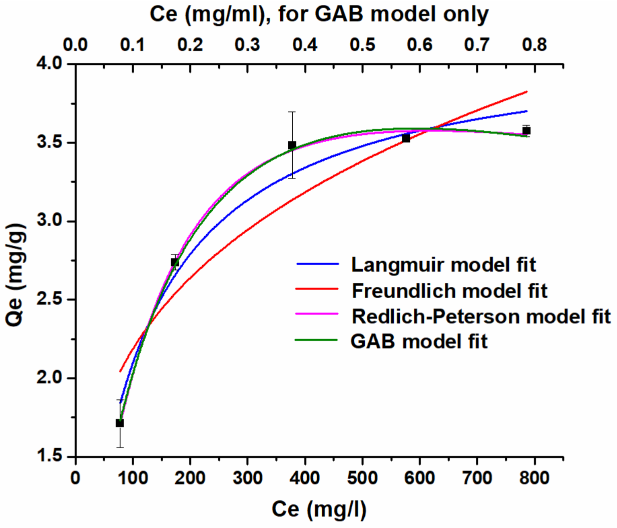

Figure S2. Fit of the four isotherm models for the experimental data of trypsin adsorption on PET MPs

Section S2. Secondary structures in trypsin bulk solution and soft corona after its incubation without or with PET and PP MPs

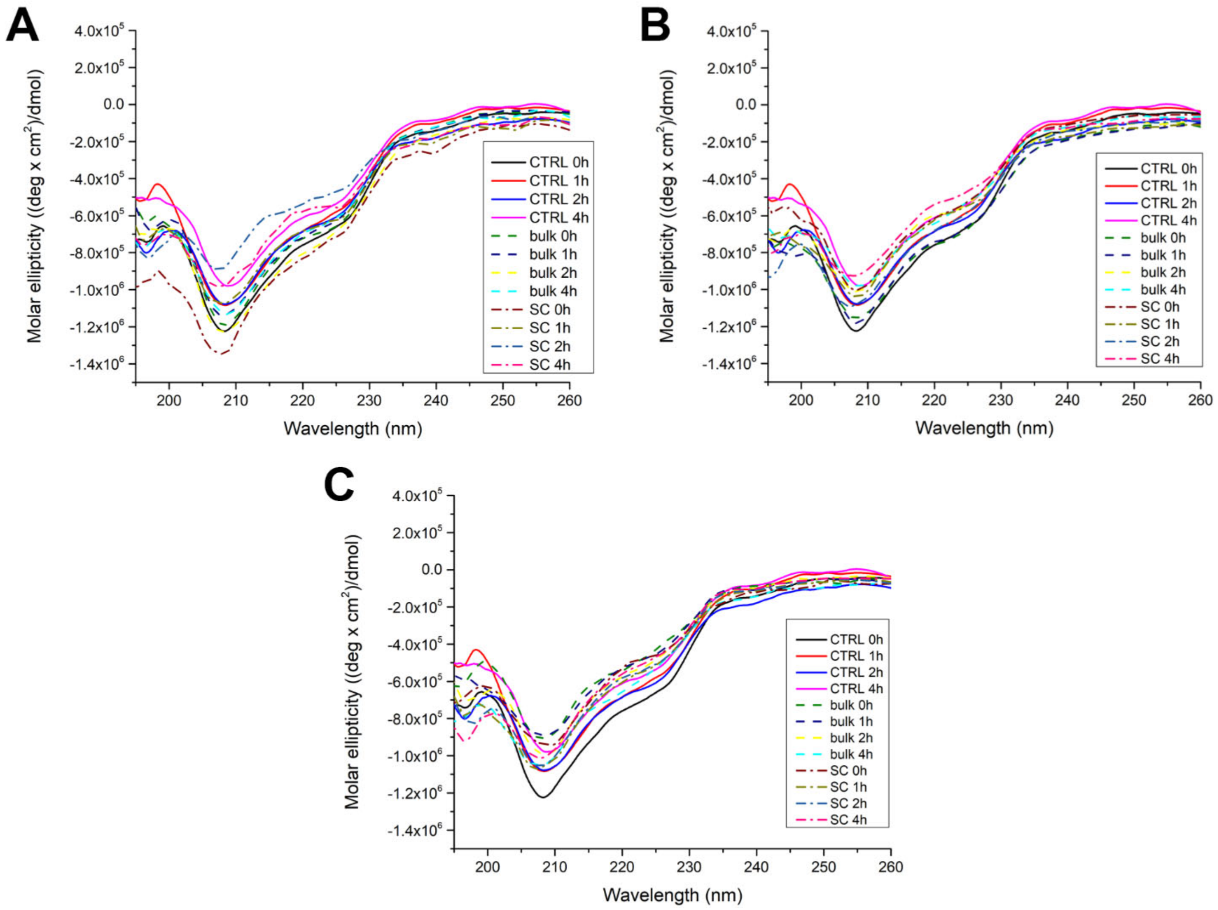

Figure S3. Far CD spectra of trypsin incubated without MPs (CTRL – control), and bulk trypsin and trypsin in SC after incubation with PET (**A**), sPP (**B**), and lPP MPs (**C**)

Table S1. Secondary structural content (%) in trypsin fractions (bulk and soft corona) after its incubation without (control) or with PET and PP MPs.

	**α-helix**	**β-sheet**	**turn**	**random**
**Control 0 h**	6.7 ± 0.4	35.7 ± 1	22.3 ± 0.3	35.3 ± 1.1
**Control 1 h**	5.1 ± 0.9	38 ± 0.7	22.3 ± 0.6	34.7 ± 0.9
**Control 2 h**	6.2 ± 0.4	37.3 ± 0.9	22.3 ± 0.2	34.2 ± 1
**Control 4 h**	4.5 ± 0.5	39.5 ± 0.7	22.1 ± 0.3	33.9 ± 0.8
**sPP bulk 0 h**	7.4 ± 0.3	36.1 ± 0.2	22.1 ± 0.1	34.5 ± 0.3
**sPP bulk 1 h**	7.6 ± 0.3	36.3 ± 0.7	22.0 ± 0.1	34.2 ± 0.8
**sPP bulk 2 h**	5.3 ± 0.4	39.1 ± 0.3	22.2 ± 0.2	33.5 ± 0.4
**sPP bulk 4 h**	5.5 ± 0.4	38.6 ± 0.6	21.9 ± 0.2	34.1 ± 0.6
**sPP SC 0 h**	5.2 ± 0.7	39.6 ± 1.0	22.0 ± 0.6	33.3 ± 1.0
**sPP SC 1 h**	5.8 ± 0.4	38.8 ± 0.1	21.9 ± 0.4	33.5 ± 0.3
**sPP SC 2 h**	5.8 ± 0.6	36.6 ± 0.5	22.5 ± 0.4	35.1 ± 0.7

**sPP SC 4 h**	4.6 ± 0.4	39.8 ± 0.3	22.1 ± 0.2	33.4 ± 0.5
**lPP bulk 0 h**	4.2 ± 0.5	40.9 ± 1.5	22.1 ± 0.5	32.7 ± 1.4
**lPP bulk 1 h**	4.5 ± 0.1	41.0 ± 0.8	21.7 ± 0.2	32.8 ± 0.6
**lPP bulk 2 h**	4.8 ± 0.4	40.1 ± 0.3	21.9 ± 0.3	33.2 ± 0.3
**lPP bulk 4 h**	5.8 ± 0.5	38.1 ± 1.1	22.1 ± 0.2	34.1 ± 1.3
**lPP SC 0 h**	4.3 ± 0.7	40.2 ± 0.5	22.5 ± 0.6	33.0 ± 0.7
**lPP SC 1 h**	5.3 ± 0.8	39.0 ± 1.3	22.0 ± 0.8	33.7 ± 1.3
**lPP SC 2 h**	5.2 ± 0.9	39.2 ± 0.8	21.8 ± 0.8	33.7 ± 0.7
**lPP SC 4 h**	4.6 ± 0.5	39.0 ± 0.8	22.0 ± 0.4	34.3 ± 0.8
**PET bulk 0 h**	6.5 ± 0.4	36.8 ± 0.5	22.1 ± 0.3	34.6 ± 0.7
**PET bulk 1 h**	6.4 ± 0.7	37.2 ± 0.7	22.1 ± 0.3	34.4 ± 1.2
**PET bulk 2 h**	7.5 ± 0.3	35.7 ± 0.4	22.3 ± 0.1	34.6 ± 0.5
**PET bulk 4 h**	5.8 ± 0.3	36.6 ± 0.6	22.4 ± 0.1	35.3 ± 0.7
**PET SC 0 h**	8.1 ± 0.8	34.5 ± 0.3	22.1 ± 0.6	35.4 ± 0.3
**PET SC 1 h**	6.6 ± 0.5	38.0 ± 0.8	22.0 ± 0.2	33.4 ± 0.5
**PET SC 2 h**	5.1 ± 0.2	40.7 ± 1.1	21.5 ± 0.4	32.8 ± 0.9
**PET SC 4 h**	5.4 ± 1.3	39.8 ± 0.6	22.2 ± 0.7	32.6 ± 1.1

Section S3.

S3.1. Preparation of PET and PP MPs

Materials

Food grade polypropylene (PP, CAS: 9003-07-0) and polyethylene terephthalate (PET, CAS: 25038-59-9) granules were kindly provided by PlasticsEurope.

Milling

To produce PP powders, pre-cooled PP granules (20 g, -70 °C) were mixed with ethanol (40 mL, 96%, v/v) and dry ice in the cryomilling container of the GM 200 knife mill (Retsch GmbH, Haan, Germany). Grinding was carried out with the stainless-steel accessories at different speeds from 4000 to 10000 rpm for 3 to 5 min at 30 s intervals to prevent overheating. Three main stages were: i) hit mode (backwards rotation and initial comminution with the blunt side of the knife) for two cycles of 30 s at 4000 rpm, ii) cut mode for two cycles of 30 s at 5000 rpm, and iii) adding of the volume reduction lid (increased contact time of the milling good with the knifes) and fine milling for two cycles of 30 s at 5000, 6500,

7500, and 8500 rpm, and three cycles of 60 s each at 10000 rpm. Dry ice was added periodically, to keep the temperature below the glass transition temperature (T_g, -20 to 0 °C) of PP [65].

Powders of PET were produced using the ultra-centrifugal mill ZM 200 (Retsch GmbH, Haan, Germany) with an 80 µm ring sieve with trapezoid holes (03.647.0465) and cyclone accessories. Similarly to Ducoli et al. (2022) [67], the PET granules (10 g) were first pre-cooled in liquid nitrogen for 30 min to keep the temperature below the T_g (70 to 80 °C) of PET [66]. Granules were added stepwise with a stainless-steel spoon and milled at 18000 rpm.

Size fractionation

The dry powders were collected by rinsing the milling containers with ethanol (96%, v/v) and collecting the suspensions in glass beakers. Initial size separation of the PP suspension was achieved by sieving it through a stack of stainless-steel mesh sieves (63 µm, 180 µm, 500 µm) on a collecting pan, where the widest mesh was at the top. A vibratory sieve shaker (AS 200–digit CA, Retsch GmbH, Haan, Germany) was used for agitation in interval mode for 7 min with an amplitude of 1.50 mm. Due to electrostatic charging of the PP microplastics, the different size fractions were collected in glass beakers by rinsing the sieves with ethanol (96%, v/v). Sieving the PET particles was not necessary because only particles < 80 µm were able to pass the ring sieve of the mill. Particles were collected with ethanol, as was done for PP.

The pre-fractionated PP suspensions were then vacuum filtered through a 10 µm nylon net filter (NY1004700, Merck Millipore KGaA, Billerica, USA), to remove fines and excess ethanol, and brought to dryness using a rotary evaporator (Heidolph Instruments GmbH & Co. KG, Schwabach, Germany). Similarly, the PET fraction was filtered through a 0.8 µm nylon membrane (WHA7408004, Whatman plc, Maidstone, UK) and dried completely before further use.

References

[65] Maddah, H.A. Polypropylene as a Promising Plastic: A Review. *American Journal of Polymer Science,* **2016**, *6*, 1-11. doi:10.5923/j.ajps.20160601.01.
[67] Ducoli, S.; Federici, S.; Nicsanu, R.; Zendrini, A.; Marchesi, C.; Paolini, L.; Radeghieri, A.; Bergese, P.; Depero, L.E. A different protein corona cloaks "true-to-life" nanoplastics with respect to synthetic polystyrene nanobeads. *Environ. Sci.: Nano* **2022**, *9*, 1414-1426. doi: 10.1039/d1en01016f.
[66] Thomsen, T.B.; Hunt, C.J.; Meyer, A.S. Influence of substrate crystallinity and glass transition temperature on enzymatic degradation of polyethylene terephthalate (PET). *N. Biotechnol.* **2022**, *69*, 28-35. doi: 10.1016/j.nbt.2022.02.006.

S3.2. Characterization of PET and PP MP

a) Determination of MPs particle size distribution using dynamic laser diffraction

Appropriate amounts of dry powders were weighed-in into a glass vial and mixed with a Tween®80 (Sigma-Aldrich, St. Louis, Missouri, United States) solution (5%, w/w) to obtain concentrations of 1 mg/mL (PET), 5 mg/mL (sPP), and 10 mg/mL (lPP) depending on their size and with respect to their density. Tween®80 solution was previously filtered through a 0.22 μm PTFE syringe filter. The non-ionic surfactant Tween®80 is needed to obtain well-dispersed samples and is only used for quality control. The particle size distribution was then measured via laser diffraction (LD) with a Mastersizer3000 (Malvern Panalytical Ltd., Malvern, UK) and the HydroSV measurement cell. The cell was filled with 5.5 mL of a 0.3% Tween®80 solution and samples were added stepwise after the background measurement until an obscuration level (optical density value) of 10-15% was achieved. Average particle size distributions of 10 consecutive measurements were calculated and reported. Size distribution is expressed through volume density (**Figure S4).** The median size of PET particles is 55 μm and 62.8 μm, of sPP 117 μm and of lPP 326 μm (**Table S2**).

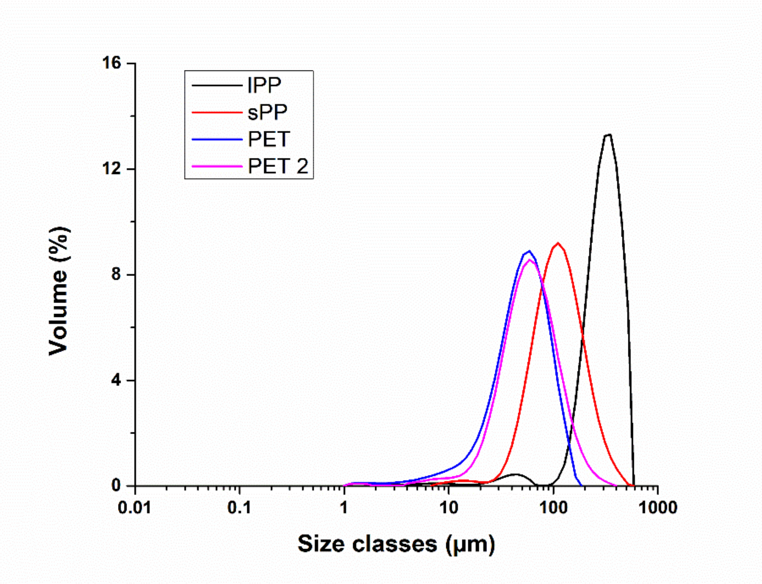

Figure S4. Size distribution of MPs expressed as volume density

Table S2. Lower (Dv10), median (Dv50), and upper (Dv90) size of MP particles determined by laser diffraction.

	D10 (μm)	D50 (μm)	D90 (μm)
PET	21.6	55.0	106
PET 2	28.1	62.8	134
sPP	57.7	117	238
lPP	187	326	494

b) Characterization of MPs by microFTIR

Fourier Transform Infrared Spectroscopy was conducted to identify polymer composition. The spectra were matched against a reference library to determine polymer identity and match percentage. The analysis was performed using a **Thermo Fisher Nicolet iN10** micro-FTIR (microscope coupled with an infrared spectrophotometer, Thermo Fisher scientific, Waltham, USA). The ultra-fast mapping feature was utilized to scan gold-coated slides, onto which the microplastic samples were transferred. This approach ensured high spatial resolution and efficient identification of polymer types with region from 3301.66 to 750.00.cm^{-1}.

Table S3. Polymer identification and match percentage in analyzed samples

Sample name	Match [%]
PET	90.21
PET 2	84.62
sPP	86.55
lPP	89.12

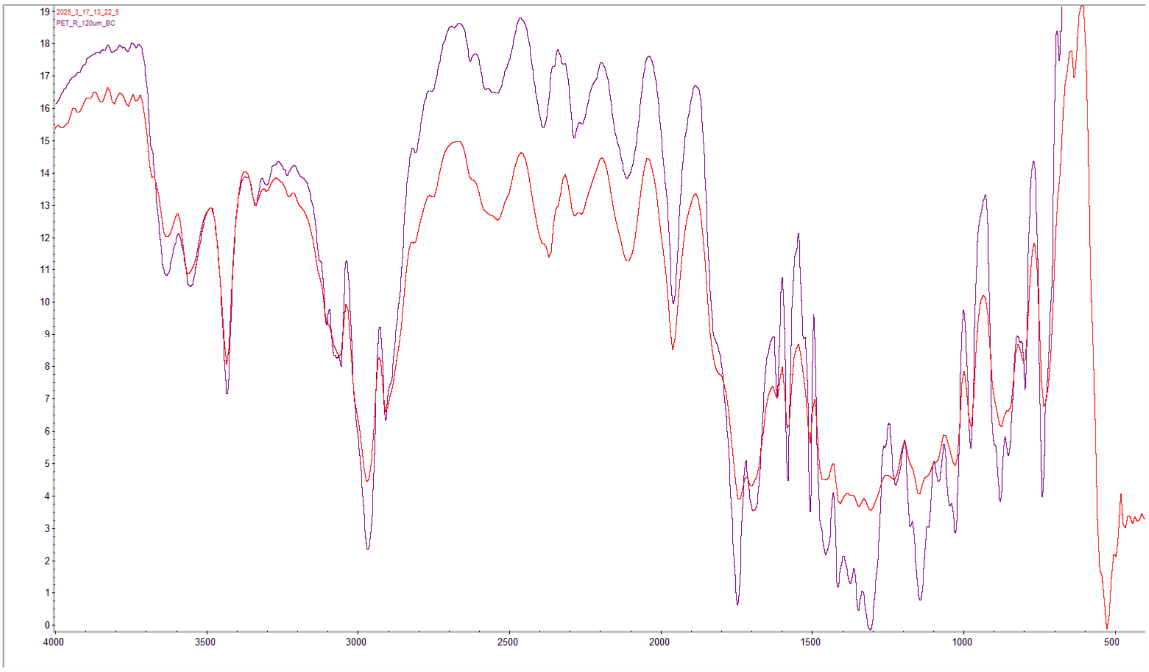

Figure S5. FTIR spectrum of PET sample (red) and PET from library (violet).

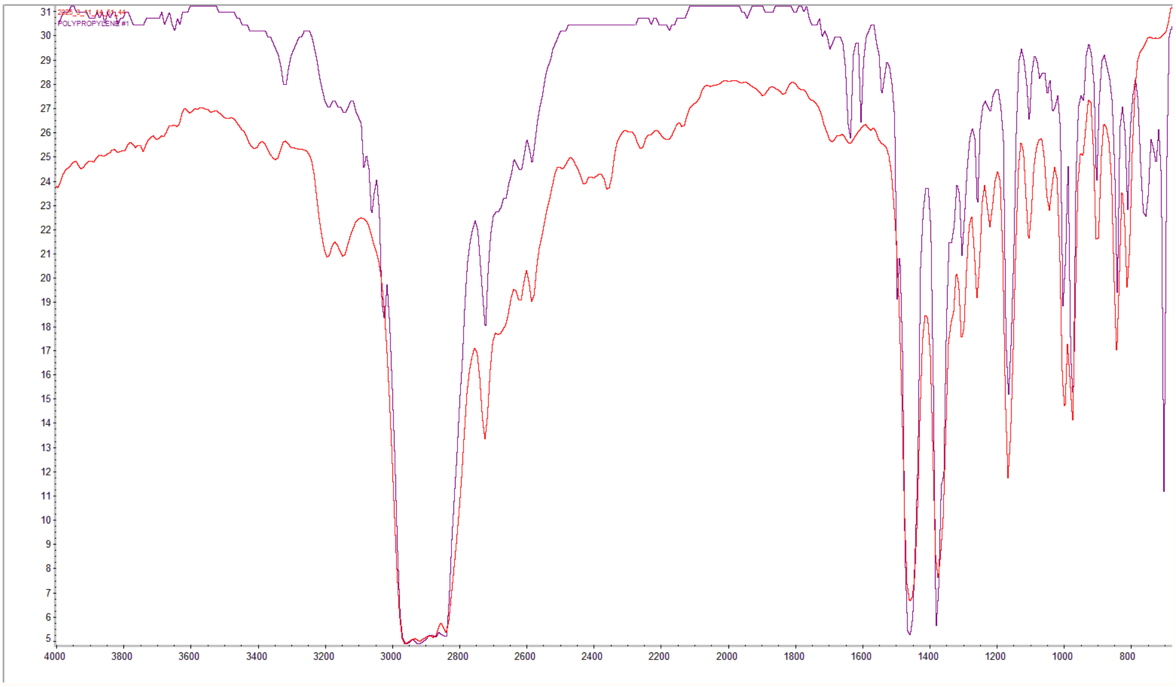

Figure S6. FTIR Spectrum of sPP sample (red) and PP from library (violet).

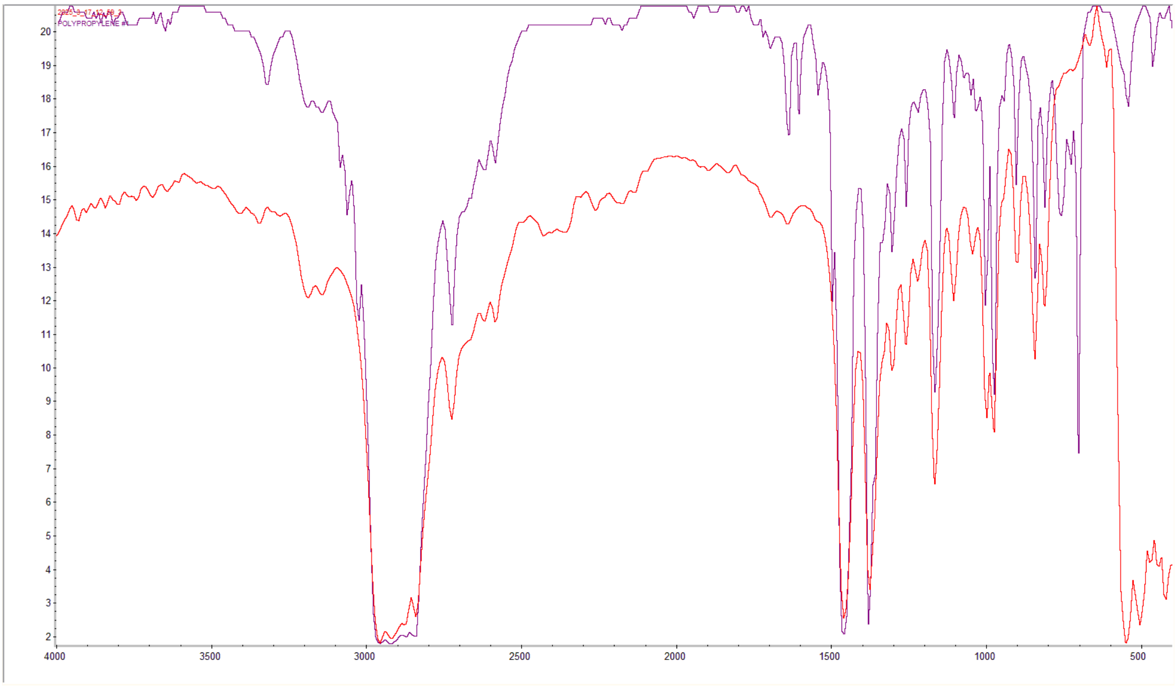

Figure S7. FTIR Spectrum of lPP sample (red) and PP from library (violet).

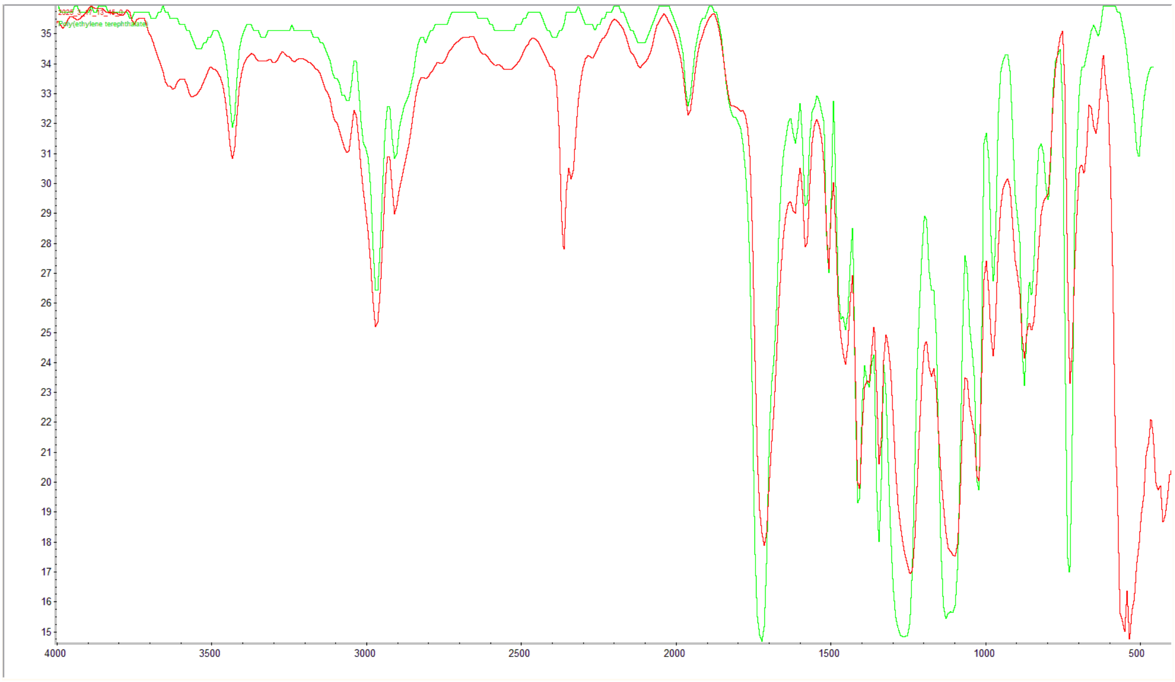

Figure S8. FTIR Spectrum of PET 2 sample (red) and PET from library (green)

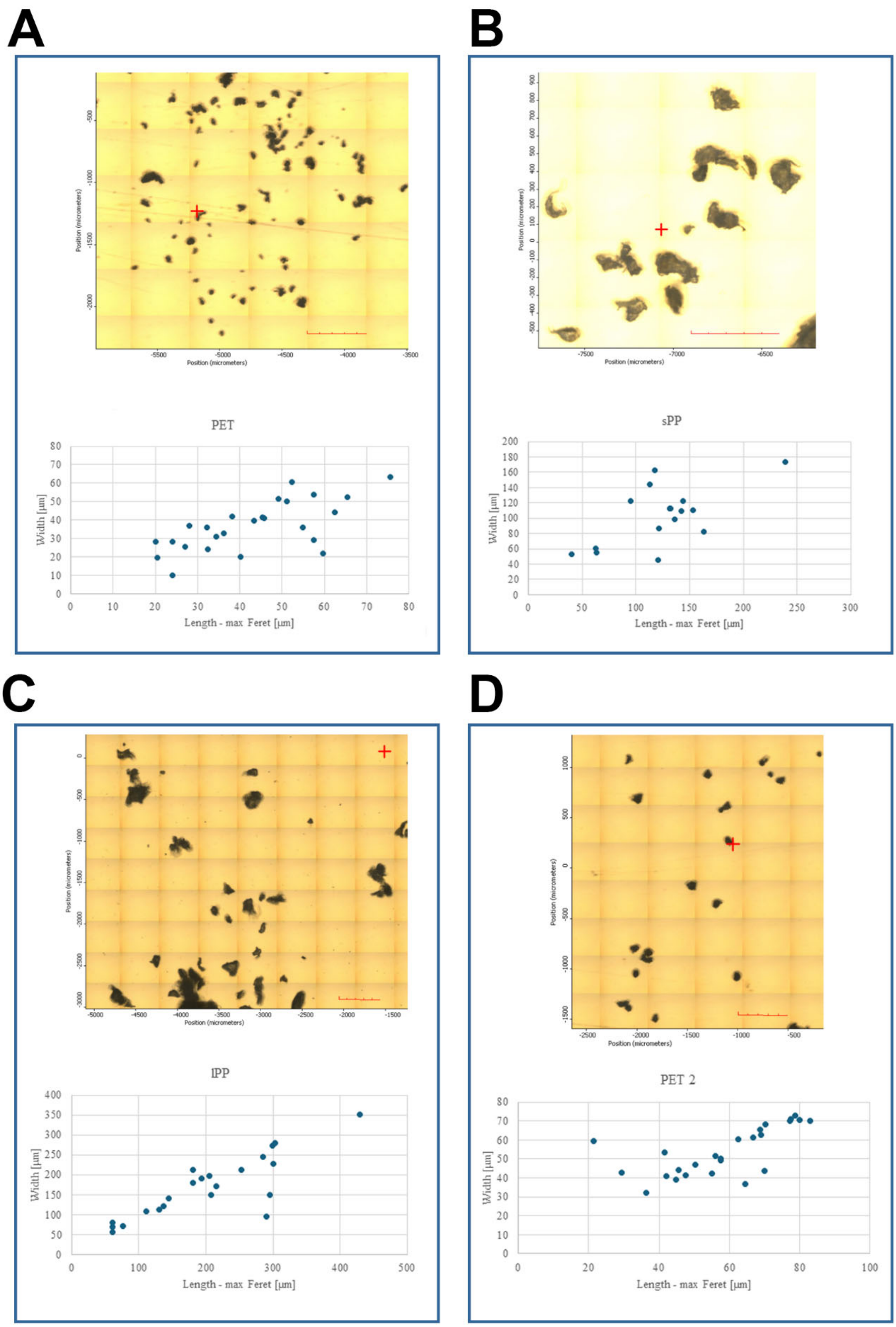

Figure S9. Microscopic images with scatter plots of the particle size distribution of PET (**A**), sPP (**B**), lPP (**C**), and PET 2 (D).

Section S4. Composition of simulated intestinal fluid (SIF) buffer

Table S4. Composition of SIF according to Brodkorb et al. (2019)[68]

Component	Final concentration (mM)
**KCl**	6.8
**KH_2PO_4**	0.8
**$NaHCO_3$**	85
**NaCl**	38.4
**$MgCl_2$**	0.33
**$CaCl_2$**	0.6

[68] Brodkorb, A.; Egger, L.; Alminger, M.; Alvito, P.; Assunção, R.; Ballance, S.; Bohn, T.; Bourlieu-Lacanal, C.; Boutrou, R.; Carrière, F.; et al. INFOGEST Static in Vitro Simulation of Gastrointestinal Food Digestion. *Nat. Protoc.* **2019**, *14*, 991–1014, doi:10.1038/s41596-018-0119-1.

Section S5. Preparation of beef meat extract

Beef round meat was fresh from a local store and stored overnight at -20°C. Raw meat extract was prepared using a previously described protocol [74]. Briefly, one piece of frozen beef (10 g) was homogenized with 30 mL of simulated salivary fluid (SSF) with an ULTRA-TURRAX® homogenizer (T25 digital, IKA, Staufen, Germany). The obtained extract was incubated with gentle mixing on a mini rocker-shaker (MR-1, Biosan, Riga, Latvia) for 1 h at 4°C and subsequently centrifuged at 4500 g for 30 min at 4°C (5804R, Eppendorf, Hamburg, Germany). The supernatant was collected and filtered through a 0.22 μm filter, and protein content was measured with a BCA assay (Pierce, Rockford, IL, USA). The protein concentration was 13.55 ± 0.51 mg/mL.

References

[74] Apostolovic, D.; Tran, T.A.T.; Hamsten, C.; Starkhammar, M.; Cirkovic Velickovic, T.; van Hage, M. Immunoproteomics of processed beef proteins reveal novel galactose-a-1,3-galactose-containing allergens. *Allergy,* **2014**, *69*, 1308–1315. doi:10.1111/all.12462

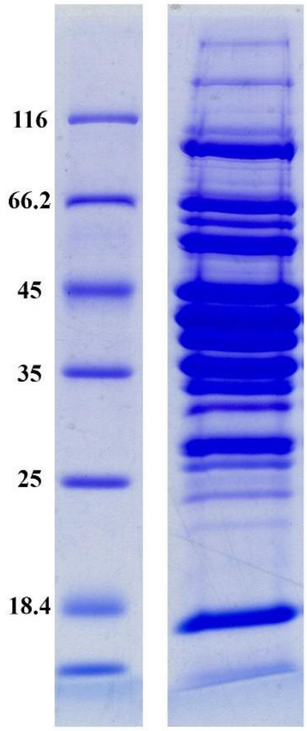

Figure S10. Protein profile of beef red meat extract on 12% acrylamide gel under reducing conditions.

Section S6. Immunoblot detection of α--Gal epitope

Immunoblots for the detection of α-Gal epitope in digested beef meat proteins were performed using commercial human monoclonal IgM antibodies to α-Gal (Abcam, Cambridge, UK) Proteins resolved by SDS PAGE were transferred to nitrocellulose (NC) membranes using an EBU-4000 Semi-Dry Blotting System (Thermo Fisher Scientific; Waltham, Massachusetts, United States). Membranes (Nitrocellulose, Whatman GmbH, Dassel, Germany), were blocked with 1% human serum albumin (made from 20% solution, Takeda, Tokyo, Japan) in Tris-buffered saline with 0.1% Tween®20 detergent (tTBS) for 1 h. After blocking, the membranes were incubated with a 1:5000 dilution of anti-α-Gal antibodies in 0.1% HSA in tTBS overnight at 4℃. After the incubation with primary antibodies, membranes were washed 3 times for 5 minutes with tTBS. Goat polyclonal antibodies to human IgM labeled with alkaline phosphatase (Abcam, Cambridge, UK) in 0.1% HSA in tTBS diluted 1:5000 times were added as secondary antibodies for 1 h at room temperature. Membranes were washed 2 times for 5 min with tTBS and one time with TBS. Protein bands were visualized using BCIP (5-Bromo-4-chloro-3-indolyl phosphate p-toluidine salt, SERVA Electrophoresis GmbH, Heidelberg, Germany) and NBT (Nitro blue tetrazolium, SERVA Electrophoresis GmbH, Heidelberg, Germany).
